# Multi-strain probiotics alleviate loperamide-induced constipation by adjusting the microbiome, serotonin, and short-chain fatty acids in rats

**DOI:** 10.3389/fmicb.2023.1174968

**Published:** 2023-06-02

**Authors:** Jin-Ju Jeong, Raja Ganesan, Yoo-Jeong Jin, Hee Jin Park, Byeong Hyun Min, Min Kyo Jeong, Sang Jun Yoon, Mi Ran Choi, Jieun Choi, Ji Hyun Moon, Uigi Min, Jong-Hyun Lim, Do Yup Lee, Sang Hak Han, Young Lim Ham, Byung-Yong Kim, Ki Tae Suk

**Affiliations:** ^1^Institute for Liver and Digestive Disease, Hallym University, Chuncheon, Republic of Korea; ^2^R&D Center, Chong Kun Dang Healthcare, Seoul, Republic of Korea; ^3^Department of Agricultural Biotechnology, Center for Food and Bioconvergence, Research Institute of Agricultural and Life Sciences, Seoul National University, Seoul, Republic of Korea; ^4^Department of Pathology, Hallym University College of Medicine, Chuncheon, Republic of Korea; ^5^Department of Nursing, Daewon University College, Jecheon, Republic of Korea

**Keywords:** constipation, probiotics, microbiome, serotonin, SCFAs

## Abstract

Constipation is one of the most common gastrointestinal (GI) disorders worldwide. The use of probiotics to improve constipation is well known. In this study, the effect on loperamide-induced constipation by intragastric administration of probiotics Consti-Biome mixed with SynBalance® SmilinGut (*Lactobacillus plantarum* PBS067, *Lactobacillus rhamnosus* LRH020, *Bifidobacterium animalis* subsp. *lactis* BL050; Roelmi HPC), *L. plantarum* UALp-05 (Chr. Hansen), *Lactobacillus acidophilus* DDS-1 (Chr. Hansen), and *Streptococcus thermophilus* CKDB027 (Chong Kun Dang Bio) to rats was evaluated. To induce constipation, 5 mg/kg loperamide was intraperitoneally administered twice a day for 7 days to all groups except the normal control group. After inducing constipation, Dulcolax-S tablets and multi-strain probiotics Consti-Biome were orally administered once a day for 14 days. The probiotics were administered 0.5 mL at concentrations of 2 × 10^8^ CFU/mL (G1), 2 × 10^9^ CFU/mL (G2), and 2 × 10^10^ CFU/mL (G3). Compared to the loperamide administration group (LOP), the multi-strain probiotics not only significantly increased the number of fecal pellets but also improved the GI transit rate. The mRNA expression levels of serotonin- and mucin-related genes in the colons that were treated with the probiotics were also significantly increased compared to levels in the LOP group. In addition, an increase in serotonin was observed in the colon. The cecum metabolites showed a different pattern between the probiotics-treated groups and the LOP group, and an increase in short-chain fatty acids was observed in the probiotic-treated groups. The abundances of the phylum *Verrucomicrobia*, the family *Erysipelotrichaceae* and the genus *Akkermansia* were increased in fecal samples of the probiotic-treated groups. Therefore, the multi-strain probiotics used in this experiment were thought to help alleviate LOP-induced constipation by altering the levels of short-chain fatty acids, serotonin, and mucin through improvement in the intestinal microflora.

## Introduction

Constipation is a common gastrointestinal (GI) disorder, and its prevalence is estimated to be 16% worldwide (Forootan et al., 2018). This symptom was reported more often in women than in men and in elderly people than in young people (Faigel, 2002). Various causes of constipation are known, but it can result from intestinal obstruction, lack of exercise, low fiber intake, or personal factors (Bosaeus, 2004). Laxatives that induce diarrhea are generally used for the treatment of constipation, and types include bulk-forming agents, chloride-channel activators, emollients, hyperosmotics, lubricants, saline laxatives, serotonin antagonists, and stimulants. However, they may have adverse effects such as diarrhea, cramping, and headache (Ambizas and Ginzburg, 2007). To overcome the side effects of laxatives, modulation of the intestinal microflora with probiotics might be necessary.

Probiotics are live microorganisms that can be ingested and have beneficial effects on health. Probiotics are effective in improving various diseases, such as GI diseases, allergies, respiratory diseases, neurological and psychiatric diseases, genitourinary tract infections, and oral diseases (Jeong et al., 2022). They have a variety of mechanisms, such as competition with pathogens, secretion of bacteriocins, fatty acid production, enzymatic activities, and microbiome changes (Plaza-Diaz et al., 2019). The genera *Bifidobacterium* and *Lactobacillus* are representative bacteria that are frequently used as probiotics (Koebnick et al., 2003; Yang et al., 2008; Li et al., 2015; Wang et al., 2019). Supplementation with *Bifidobacterium* not only increased the number of bowel movements per week in patients with chronic constipation in clinical trials but also significantly improved consistency (Yang et al., 2008). *Bifidobacterium* can alleviate constipation by improving fecal fluids, propionate and butyrate, and GI transit time (Wang et al., 2019)*. Lactobacillus* improved self-diagnosed constipation severity and stool consistency in clinical trials (Koebnick et al., 2003). Similarly, *Bifidobacterium* and *Lactobacillus* relieved constipation by improving GI transit and increasing the production of fecal short-chain fatty acids (SCFAs) (Li et al., 2015). Chocolate made from *Lactobacillus* and *Streptococcus* also ameliorated constipation by increasing intestinal motility (Eor et al., 2019). Recently, the trend of using a mixture of individual probiotics is increasing. The reason is considered to be because of the possibility of synergy and additive effect rather than the use of a single strain (Kwoji et al., 2021). *Lactobacillus rhamnosus* GG (LGG) mixed with *Bifidobacterium animalis* subsp. *lactis* Bb12 was more effective in eliminating *Helicobacter pylori* and improving necrotizing enterocolitis than when LGG used alone (Myllyluoma et al., 2005; Hays et al., 2016).

Several mechanisms related to the amelioration of GI diseases by microorganisms have been identified. The gut microbiome is one of the most actively studied mechanisms in the last decade. In patients with constipation, a decrease in lactate- and butyrate-producing bacteria was observed (Dimidi et al., 2020). Butyrate belongs to the SCFA family of volatile fatty acids, with 1–6 carbon atoms attached to the aliphatic chain (Ríos-Covián et al., 2016). SCFAs were the major metabolites produced by the fermentation of anaerobic bacteria in the GI tract. Therefore, changes in the microbiome after treatment with microorganisms could be deeply related to changes in SCFAs. Among the SCFAs, acetate, butyrate, propionate, and valerate have been studied for their inhibitory effects on various diseases. Acetate is involved in cholesterol synthesis and shows a protective effect against infection by *Escherichia coli* O157:H7 (Arora and Sharma, 2011; Fukuda et al., 2011). Butyrate showed an immunoregulatory effect and was reported to have therapeutic and protective effects against distal ulcerative colitis (Böcker et al., 2003; Wang et al., 2018). SCFAs increased IL-18 production through a GPR109a-mediated pathway, which could sustain stomach homeostasis and prevent colorectal carcinogenesis (Kalina et al., 2002; Singh et al., 2014). Propionate inhibits cholesterol synthesis (Henningsson et al., 2001), and valerate has a positive effect on the pathogenesis of colitis (Yuille et al., 2018). In addition, SCFAs can stimulate the secretion of serotonin and mucin, which can improve constipation (Hatayama et al., 2007; Reigstad et al., 2015). It has been observed that serotonin is decreased in animal models of constipation, and its secretion is involved in GI motility and regulated by gut microbes (Gershon, 2004; Cao et al., 2017). Moreover, tryptophan, bile acids, lipopolysaccharide, methane, and hydrogen are known metabolites produced by microorganisms that are involved in gastrointestinal physiology (Jahng et al., 2012; Ao et al., 2013; Zelante et al., 2013; Ye et al., 2020).

Considering these factors, the use of probiotics is thought to contribute to alleviating the symptoms of constipation treatment without adverse effects caused by laxatives. In this study, the effect of multi-strain probiotics composed of *Bifidobacterium*, *Lactobacillus*, and *Streptococcus* on loperamide-induced constipation in rats was confirmed, and changes in the gut microbiome and metabolites were investigated.

## Materials and methods

### Multi-strain probiotics formulation

A multi-strain probiotics Consti-Biome containing a mixture of SynBalance® SmilinGut (*Lactobacillus plantarum* PBS067, *Lactobacillus rhamnosus* LRH020, *Bifidobacterium animalis* subsp. *lactis* BL050; Roelmi HPC), *L. plantarum* UALp-05 (Chr. Hansen), *Lactobacillus acidophilus* DDS-1 (Chr. Hansen), and *Streptococcus thermophilus* CKDB027 (Chong Kun Dang Bio) was obtained from Chong Kun Dang HealthCare (Seoul, Korea). The multi-strain probiotics Consti-Biome was adjusted to 2 × 10^10^ colony-forming unit (CFU), mixed in Dulbecco’s phosphate-buffered saline (D-PBS), vortexed for 20 min, and diluted to the concentration required for the experiment.

### Experimental animals and design

Five-week-old male Sprague Dawley (SD) rats were purchased from DooYeol Biotech (Seoul, Korea). Rats were bred under conditions of temperature of 20 ± 2°C, humidity of 55 ± 5%, and light and dark conditions for 12 h and given free access to drinking water and standard chow. After 1 week of adaptation, the rats were randomly divided into 6 groups with 7 animals in each group. They were grouped into normal control (NOR), loperamide administration (LOP), positive control (PC), low concentration multi-strain probiotics Consti-Biome administration (G1, 2 × 10^8^ CFU), medium concentration Consti-Biome administration (G2, 2 × 10^9^ CFU), and high concentration Consti-Biome administration (G3, 2 × 10^10^ CFU), respectively. As a positive control group, 5 mg/kg of constipation treatment drug called Dulcolax-S tablets (Boehringer Ingelheim, Alkmaar, Nederland) was used.

Constipation was induced by intraperitoneal administration of loperamide (Sigma, St. Louis, MO, United States) at a dose of 5 mg/kg body weight in all groups twice a day for 7 days except for NOR group. Dulcolax-S tablets and 0.5 mL of multi-strain probiotics were orally administered once a day for 14 days after induction of constipation. Body weight and food intake of the experimental animals were measured at weekly intervals. The feces of each group were collected 24 h after the bedding and feces in the cage were changed. Feces were collected at the end of the treatment. Animal sacrifice was performed by overdose of inhalational anesthesia overdose (isoflurane, Aerane; Baxter, Deerfield, IL, United States). Tissue of colon and intestine, and content of cecum were collected and stored at −80°C until required. All animal experiments were conducted in accordance with the regulations of the Animal Experimental Ethics Committee (IACUC) of Hallym University (Hallym 2021–79).

For pathological analysis of the intestine, intestinal specimens were fixed with 10% formalin, routinely embedded in paraffin; the tissue sections were processed with hematoxylin and eosin. All specimens were evaluated by the same pathologist (SHH), who was masked as to whether the specimens came from comparative or control groups. Mucosal thickness was measured by CellSens® (olympus imaging software).

### pH, water content, and intestinal motility

At the end of the multi-strain probiotics’ treatment, fecal samples were collected, then measured the number, pH, and water content, respectively. pH of diluted the fecal samples in distilled water was measured by a Ohause Starter300 pH meter (Chen et al., 2019). After drying the samples at 70°C for 24 h the water content was determined by measuring the dry weight and calculating the difference between the fresh weight, and the dry weight.

The effect of intestinal motility was measured after fasting 12 h before animal sacrifice. 1 mL of barium sulfate (1.4 g/mL; Daejung Chemicals & Metals Co. LTD., Siheung-si, Gyeonggi-do, Korea) was orally administered to the experimental animals, and after 30 min, the animals were sacrificed. Finally, by measuring the movement distance of barium sulfate in the intestine obtained from the animals, the intestinal movement rate was calculated as follows. Intestine transit rate (%) = distance moved by the barium sulfate (cm)/total intestine length (cm) × 100.

### Serotonin- (5-HT1a, 5-HT1b, Sert, Tph1, and Tph2), cytokine- (Tnf-α), and mucin- (Muc2) related genes expression in the colon

To evaluate the ability of multi-strain probiotics to regulate intestinal immunity, cytokines, mucin-related genes, and serotonin-related markers were analyzed. The colons from animals were frozen using liquid nitrogen and stored at −80°C until use. For RNA extraction, 1,000 μL of trizol reagent (Lifetechnologies, Cardisbad, CA, United States) was added to 50 mg of colon tissue and it was homogenized. 200 μL of chloroform (Sigma Aldrich, St. Louis, MO, United States) was added to the sample, left at room temperature for 5 min, and then centrifuged at 14,000 rpm, 5 min, 4°C. 1 mL of 70% cold ethanol was added to the RNA pellet and centrifuged again at 7,500 rpm, 5 min, 4°C. After removing the supernatant and drying at room temperature for 10 min, 100 μL of diethyl pyrocarbonate (DEPC) water was added and vortexed. The purity and concentration of RNA were quantified by measuring absorbance at 260 nm. 5 μg of the isolated RNA and 2.5 μL of DEPC water were put into an RT premix (Bioneer, Deajeon, Korea), and 50 μL cDNA was synthesized using the Mastercycler gradient and used as a template for PCR amplification. In the reverse transcription temperature cycle, cDNA was synthesized at 42°C for 1 h, denatured at 94°C for 5 min, and cooled at 4°C for 5 min. For PCR, 10 pg. of cDNA, 10 pg. of sense primer, antisense primer, and DEPC water were added to a PCR premix (iNtron, Seongnam, Korea) and amplification was performed using a Mastercycler gradient. Primer information for each gene is described below (Table 1).

**Table 1:** PCR primers used in this study.

Gene	Primer	Sequences 5′- > 3’
GAPDH	Forward	CCATCACCATCTTCCAGGAG
	Reverse	CCTGCTTCACCAACCTTCTTG
Muc2	Forward	GATAGGTGGCAGACAGGAGA
	Reverse	GCTGACGAGTGGTTGGTGATTG
Sert	Forward	ATCTCCTAGAACCCTGTAAC
	Reverse	GAAATGGACCTGGAGTATTG
Tph1	Forward	CACTCACTGTCTCTGAAAACGC
	Reverse	AGCCATGAATTTGAGAGGGAGG
Tph2	Forward	TAAATACTGGGCCAGGAGAGG
	Reverse	GAAGTGTCTTTGCCGCTTCTC
5-HT1a	Forward	TCCGACGTGACCTTCAGCTA
	Reverse	GCCAAGGAGCCGATGAGATA
5-HT1b	Forward	CCGGCTAACTACCTGATCGC
	Reverse	TATCCGACGACAGCCAGAAG
Tnf-α	Forward	TGCCTCAGCCTCTTCTCATT
	Reverse	GAGCCCATTTGGGAACTTCT

### Serotonin (5-HT) in colon

Fifty milligrams of colonic tissue were washed with D-PBS followed by homogenizing in D-PBS. After centrifuging for 15 min at 1500 × g, the 5-HT concentration of homogenates was measured using a 5-HT ELISA kit (Abcam, ab133053) according to the manufacturer’s instructions. Protein from the colonic tissue was quantified by the bicinchoninic assay. The result is presented as nanograms of serotonin per milliliter per microgram of protein.

### Metabolites in fecal samples

Analysis of metabolites of fecal samples was conducted followed by the previous study (Yu et al., 2021). In brief, each fecal sample was mixed with (acetonitrile:3′-dilstilled water, 1:1, volume:volume) and then homogenized with a Mixer Mill MM400 (Haan, Germany, Letsch GmbH & Co.). The homogenized samples were used for analysis of short-chain fatty acids (SCFAs) and untargeted metabolites.

SCFAs were analyzed using a Q-Exactive Plus Orbitrap MS-connected Ultimate-3,000 UPLC system. 40 μL of the supernatant of homogenized sample was mixed with 20 μL of 1-ethyl-3-(3-dimethylaminopropyl)carbodiimide hydrochloride (120 mM) dissolved in 6% pyridine solution and 200 mM 3-Nitrophenylhydrazine hydrochloride dissolved in 70% acetonitrile (ACN). These mixed samples were kept at 40°C for 30 min and diluted with 70% ACN to 2 mL (Han et al., 2015). For untargeted metabolites analysis, the cecal contents prepared above were chromatographically separated by Ultmate-3,000 UPLC system (Thermo Fisher Scientific,Waltham, MA, United States) equipped with 100 mm × 2.1 mm UPLC BEH 1.7 μm C18 column (Waters, Milford, MA, United States) and 5.0 mm × 2.1 mm UPLC BEH 1.7 μm C18 VanGuard Pre-Column (Waters, Milford, MA, United States). The mobile phase A and B was the same as used in SCFAs and flow rate was 0.300 mL/min. The MS analysis was performed on a Q-Exactive Plus Orbitrap MS instrument (Thermo Fisher Scientific, Waltham, MA, United States) in polarity switching ionization mode. Full MS scan was conducted on the metabolites (80–1,200 m/z) with resolution of 70,000 FWHM at m/z = 200 and with automatic gain control (AGC) target of 1e6 ions and maximum injection time (IT) of 100 ms. The data-dependent MS/MS analysis was performed on total pooled samples by each ionization mode (positive mode and negative mode). Data-dependent MS/MS setting was as follows: Top5 MS1 ions; resolution, 17,500 at 200 m/z; AGC target, 1e5; maximum IT, 50 ms; isolation window, 0.4 m/z; normalized collision energy (NCE), 30, 40 and 50; intensity threshold, 2e4 ions; apex trigger, 3–6 s; dynamic exclusion, 5 s.

Data acquisition and pre-processing were performed using Xcalibur software (Thermo Fisher Scientific, San José). The obtained raw data files were processed using Compound Discoverer software (version 3.2, Thermo Fisher Scientific, San José). The data processing was done following the workflow such as Select spectra, Align Retention times, Detect Unknown Compounds, Group Unknown Compounds, Fill Gaps and Search mzCloud. Align Retention Time node was set to 1 min to Maximum shift. Compound identification was done against mzCloud with criteria of 10 ppm (MS2 mass tolerance) and 70% of assignment threshold (Yu et al., 2021).

For untargeted metabolites analysis, the fecal samples prepared above were chromatographically separated by Ultmate-3,000 UPLC system (Thermo Fisher Scientific, Waltham, MA, United States) equipped with 100 mm × 2.1 mm UPLC BEH 1.7 μm C18 column (Waters, Milford, MA, United States) and 5.0 mm × 2.1 mm UPLC BEH 1.7 μm C18 VanGuard Pre-Column (Waters, Milford, MA, United States). The mobile phase A and B was the same as used in SCFAs and flow rate was 0.300 mL/min. The data were processed using Compound Discoverer software (version 3.2, Thermo Fisher Scientific, San José).

### Microbiome in fecal samples

For metagenomic sequencing analysis, genomic DNA was extracted from rat stool and library construction was performed. Briefly, gDNA was extracted using the DNeasy Power Soil kit (Qiagen, Hilden, Germany) according to the manual provided by the manufacturer. To amplify the V3 and V4 regions, sequencing libraries were prepared according to the illumine 16S Metagenomic Sequencing Library protocols. The primers used for PCR amplification were forward 5’-TCGTCGGCAGCGTCAGATGTGTATAAGAGACAGCCTACGGGNGGCWG-CAG and reverse (5’-GTCTCGTGGGCTCGGAGATGTGTATAAGAGACAGGACTACHV-GGGTATCTAATCC). Cycle conditions were 95°C for 3 min, 25 cycles at 95°C for 30 s, 55°C and 72°C for 30 s, and final extension at 72°C for 5 min. The PCR product obtained here was purified with AMPure beads (Agencourt Bioscience, Beverly, MA). The purified PCR products were amplified for final library construction using NexteraXT Indexed Primer. All PCR conditions were the same described above, except that the number of cycles was 10 times. After purification the PCR products, they were quantified by qPCR using KAPA Library Quantification kits for Illumine Sequencing platforms, and qualified with TapeStation D1000 ScreenTape (Agilent Technologies, Waldbronn, Germany). Then, sequencing was performed using the MiSeq platform (Illumina, San Diego, United States); sequence analysis of the samples was conducted by the Macrogen Inc. (Macrogen, Seoul, Korea).

For amplicon sequence variants (ASV) analysis, the Cutadapt (v3.2) program was used to remove the sequencing adapter sequence and the F/R primer sequence of the target gene region (Martin, 2011). Error-correction of the amplicon sequencing process was performed using the DADA2 (v1.18.0) package of R (v4.0.3) (Callahan et al., 2016). After combining the corrected paired-end sequences into one sequence, the chimera was removed. Using QIIME (v1.9), subsampling was applied and normalized based on the number of reads of the sample with the minimum number of reads among all samples, and the microbial community was compared and analyzed (Caporaso et al., 2010).

The mafft (v7.475) was used for multiple alignment between SVs sequences (Katoh and Standley, 2013). With information of the ASV abundance and taxonomy, a comparative analysis of various microbial communities was performed using the QIIME. Shannon index and was obtained to confirm the species diversity and uniformity of the microbial community in the samples. Alpha diversity information was confirmed through the Chao1 value. Beta diversity between samples was calculated based on weighted and unweighted UniFrac distance. PCoA visualized the relationship between samples (Rambaut, 2009; Caporaso et al., 2010).

### Statistical analysis

All data are expressed as mean ± standard error using Prism (Prism 8.0.3, GraphPad Software Inc., San Diego, United States), the significance of the system between the experimental group and the control group was verified using T-test or One way-ANOVA, and Tukey’s post-test or Dunnett’s multi comparisons test was performed. It was considered statistically significant at *p* < 0.05. Significant differences in SCFAs among six groups and in total metabolite features compared to the LOP group were determined based on Kruskal-Wallis test and Mann–whitney u-test, respectively, using Multi Experimental Viewer (MeV, TIGR).

For principal component analysis (PCA) and partial least squares-discriminant analysis (PLS-DA) analysis of metabolites, SIMCA 17 (Umetrics AB, Umea, Sweden) was used.

## Results

### Food intake and body weight

When constipation was induced by injecting loperamide into 6-week-old experimental animals for 7 days, their body weight and food intake were not significantly different in any of the groups compared with the normal control group (NOR) (Table 2). In detail, no significant changes in body weight and food intake were observed before induction of constipation (1 week), after induction of constipation with loperamide (LOP) (2 weeks), and after administration of multi-strain probiotics (G1: 2 × 10^8^ CFU; G2: 2 × 10^9^ CFU; and G3: 2 × 10^10^ CFU) or Dulcolax-S tablets (PC) (3 and 4 weeks). These data indicate that loperamide-induced constipation has no effect on body weight or appetite.

**Table 2:** Body weight and food intake by week of experimental animals.

	NOR*	LOP	PC	G1	G2	G3
1 week						
Body weight (g)	113.9 ± 2.3**	113.8 ± 1.5	113.4 ± 1.3	112.9 ± 1.0	112.7 ± 2.1	112.7 ± 2.7
2 weeks						
Body weight (g)	163.2 ± 3.3	156.56 ± 2.8	154.3 ± 3.4	154.3 ± 3.0	153.6 ± 3.4	155.2 ± 26
Feed intake (g/day)	16.3 ± 0.2	14.5 ± 0.2	14.0 ± 0.4	14.8 ± 0.4	14.1 ± 0.2	16.0 ± 0.2
3 weeks						
Body weight (g)	190.9 ± 3.9	184.0 ± 4.0	180.9 ± 3.2	182.3 ± 3.3	181.3 ± 5.3	188.7 ± 4.3
Feed intake (g/day)	15.4 ± 0.7	15.9 ± 0.1	15.6 ± 0.1	16.6 ± 0.3	16.3 ± 0.3	17.1 ± 0.3
4 weeks						
Body weight (g)	248.3 ± 12.6	255.4 ± 5.2	247.3 ± 5.0	254.0 ± 4.0	248.0 ± 7.1	255.3 ± 5.6
Feed intake (g/day)	22.2 ± 0.2	22.2 ± 0.2	21.1 ± 0.1	22.5 ± 0.4	22.5 ± 0.4	21.9 ± 0.3

*NOR, normal control; LOP, loperamide administration; PC, positive control; G1, low concentration multi-strain probiotics administration (2 × 10^8^ CFU); G2, medium concentration multi-strain probiotics (2 × 10^9^ CFU), and G3, high concentration multi-strain probiotics (2 × 10^10^ CFU) groups.

**Data are mean ± SEM of 7 animals.

### Improvement of constipation-related indicators by the multi-strain probiotics

**Figure 1:**
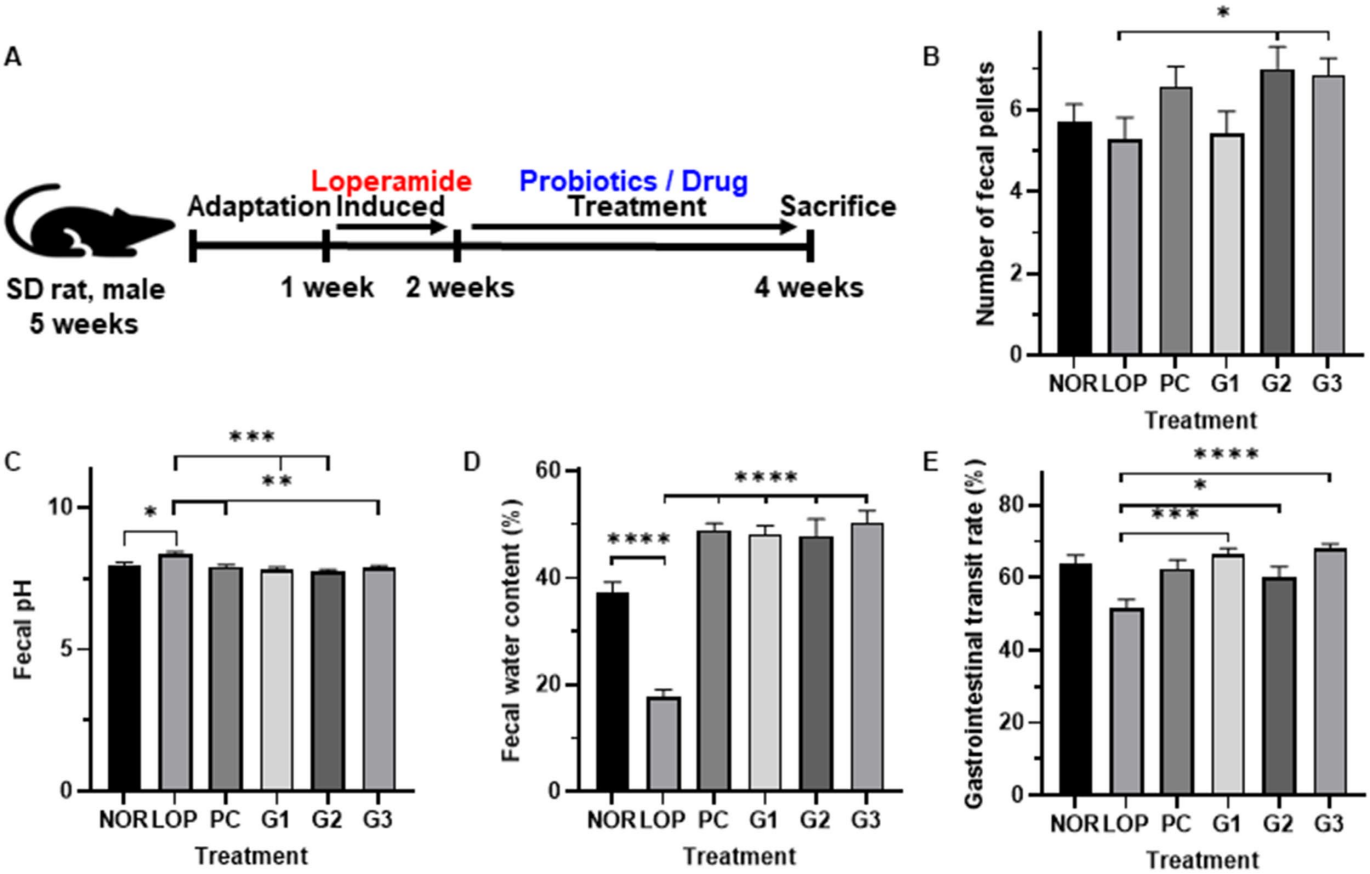
Changes in indicators related to constipation by multi-strain probiotics Consti-Biome. **(A)** Showed experimental scheme. **(B)**; number of fecal pellets, **(C)**; Fecal pH, **(D)**; Fecal water content, **(E)**; gastrointestinal transit rate of normal control (NOR), loperamide administration (LOP), positive control (PC), low concentration multi-strain probiotics administration (G1, 2 × 10^8^ cfu/mL), medium concentration multi-strain probiotics administration (G2, 2 × 10^9^ cfu/mL), and high concentration probiotic administration (G3, 2 × 10^10^ cfu/mL) groups. Data were collected 2 weeks after treatment. Data are mean ± SEM of 7 animals. *p < 0.05, **p < 0.01, *** *p* < 0.001, **** *p* < 0.0001, significantly different from the LOP as per one-way ANOVA with the Tukey’s multiple comparisons test.

Figure 1A shows the overall animal experimental design. The average number of fecal pellets was 6 in the NOR, 5 in the LOP, 7 in the PC, 5 in the G1, and 7 in the G2 and G3 groups. However, compared to the LOP, there was no significant difference in the PC and G1 groups, and a significant difference was observed in the G2 and G3 groups (Figure 1B). In both groups, the number of fecal was increased about 1.3 times compared to LOP. In all probiotic groups, the pH and water content of the feces were improved similarly to PC and significantly improved compared with the LOP (Figure 1C). The water content reduced by constipation was increased by 2.7- to 2.9-fold and 2.8-fold in the G1-3 and PC groups, respectively, compared to the LOP group, which had a water content of 17.6%, and improved similarly to the NOR group (Figure 1D). In particular, the gastrointestinal (GI) transit rate, one of the most important indicators of constipation, was confirmed to be 63.8% on average in the NOR, 51.5% in the LOP, 62.4% in the PC, 66.6% in the G1, 60.1% in the G2, and 68.2% in the G3 group. In G3, which had the highest increase among all groups, it increased by about 1.3 times compared to LOP. The GI transit rate was significantly decreased in the LOP group compared to the NOR group and was significantly increased compared to the LOP group in both the PC and probiotic administration groups (Figure 1E).

Induction of constipation with loperamide is known to decrease the thickness of the intestinal mucosa and to delay the movement of contents in the intestine (Sin et al., 2010). Although, there was no significant difference in the mucosal thickness or length in all groups, both showed a tendency to increase in the multi-strain probiotics-treated groups G1, G2, and G3 compared to LOP (Supplementary Figure S1).

### Serotonin-, cytokine-, and mucin-related mRNA expression

**Figure 2:**
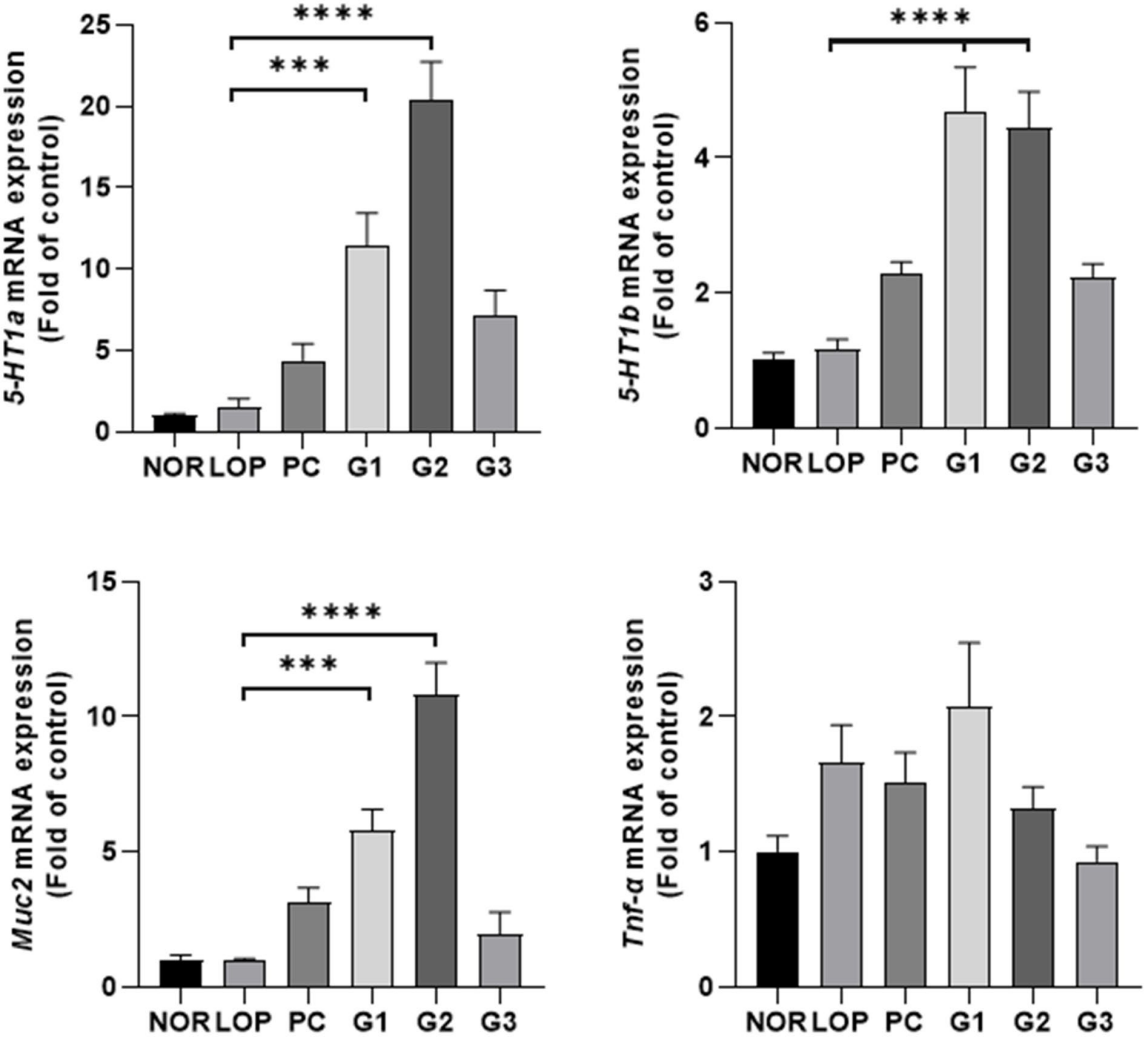
mRNA expression related to serotonin, cytokine, and mucin according to constipation induction and multi-strain probiotics Consti-Biome treatment. mRNA expression level in the colon of normal control (NOR), loperamide administration (LOP), positive control (PC), low concentration multi-strain probiotics administration (G1, 2 × 10^8^ cfu/mL), medium concentration multi-strain probiotics administration (G2, 2 × 10^9^ cfu/mL), and high concentration probiotic administration (G3, 2 × 10^10^ cfu/mL) groups were measured 2 weeks after the multi-strain probiotics’ treatment. Data are mean ± SEM of 7 animals. *p < 0.05, **p < 0.01, ***p < 0.001, ****p < 0.0001, significantly different from the LOP as per one-way ANOVA with the Tukey’s post-test.

Secretion of serotonin can be stimulated in the intestinal tract, and serotonin relay peristalsis of the intestinal tract occurs (Park, 2011). Administration of multi-strain probiotics significantly increased the expression of serotonin-related genes (5-HT1a and 5-HT1b) and mucin-related gene (Muc2) in the colons of experimental animals (Figure 2). 5-HT1a was increased approximately 8-, 14-, and 7.1-fold in the G1, G2, and G3 groups, respectively, compared with LOP. The PC group also showed a tendency to increase approximately 3 times, but there was no statistically significant change in the PC and G3 groups compared to LOP. 5-HT1b was increased approximately 4 times in both the G1 and G2 groups and 2 times in the G3 group compared with LOP (Figure 2). Similar to 5-HT1a, the PC group showed a tendency to increase approximately 2-fold, but there was no significant change in the PC and G3 groups compared to LOP. *Sert*, *Tph1*, and *Tph2*, which are also related to serotonin, showed an increasing trend in the probiotic treatment groups, but a significant difference was not observed compared to the LOP group (Supplementary Figure S2). Mucin, which plays a role in protecting the intestine, is increased by laxatives (Kim et al., 2018). The expression of muc2, an oligomeric mucus/gel-forming protein coding gene, increased approximately 6-fold and 11-fold in the G1 and G2 groups, respectively, compared to the LOP (Figure 2). Tnf-α, which is known to be increased in inflammatory diseases, showed a tendency to increase in all constipation-induced groups. However, it tended to recover in the PC, G2, and G3 groups (Figure 2). This became evident with increasing probiotic concentrations. Taken together, the multi-strain probiotics used in this experiment have the effect of improving constipation by secreting serotonin and mucin and have anti-inflammatory potential.

### Serotonin in the colon

**Figure 3:**
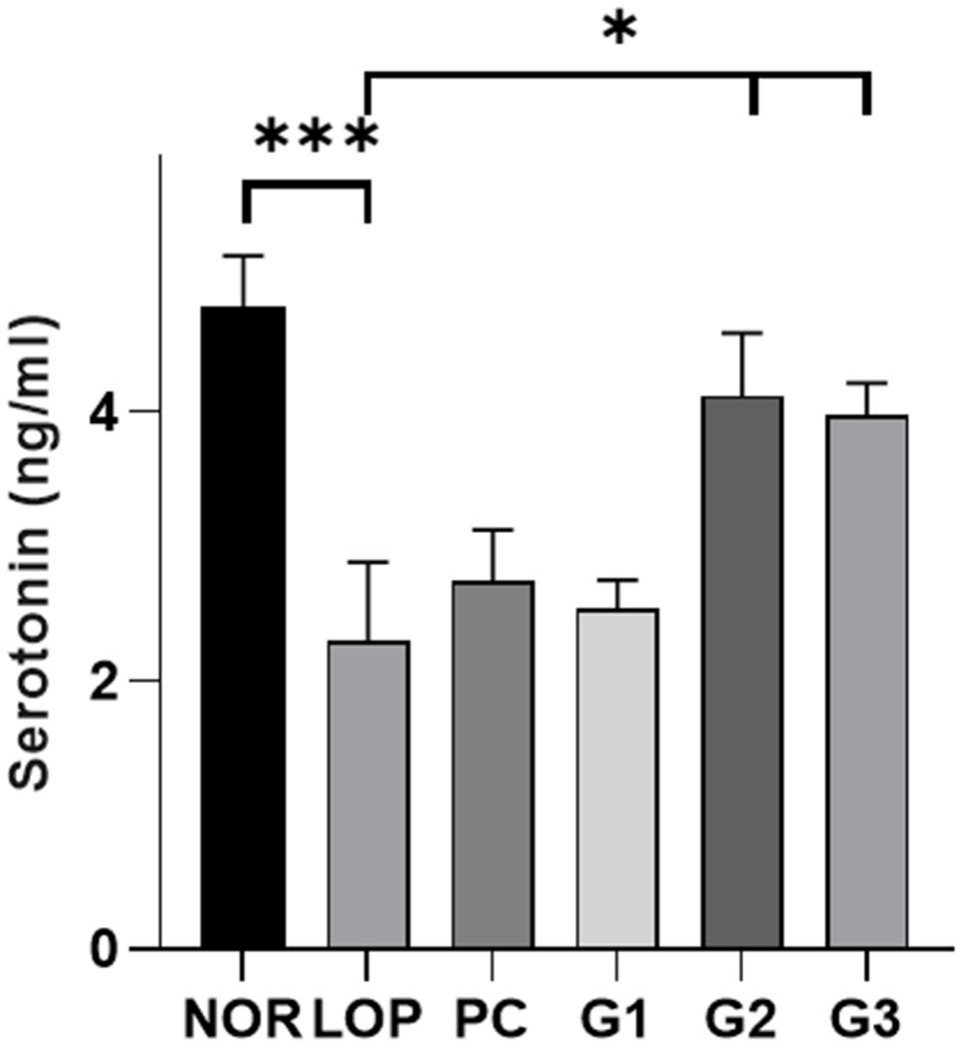
Serotonin (5-HT) levels (ng/ml) in colon samples. Colonic serotonin level of normal control (NOR), loperamide administration (LOP), positive control (PC), low concentration multi-strain probiotics administration (G1, 2 × 10^8^ cfu/mL), medium concentration multi-strain probiotics administration (G2, 2 × 10^9^ cfu/mL), and high concentration probiotic administration (G3, 2 × 10^10^ cfu/mL) groups were measured 5-HT ELISA kit. Data are mean ± SEM of 5 animals. *p < 0.05, **p < 0.01, significantly different from the LOP as per one-way ANOVA with the Dunnett’s multi comparisons test.

Serotonin is known to play a pivotal role in gastrointestinal disorders, particularly lower functional gastrointestinal disorders (Camilleri, 2009). It is related to intestinal peristalsis and intestinal motility (Costedio et al., 2007). The multi-strain probiotics used in this experiment were found to significantly increase serotonin-related genes in the colons of experimental animals. Therefore, the amount of serotonin secreted in the colon was measured. In the NOR group, an average of 4.8 ng/mL serotonin was secreted. In the LOP group, a significant decrease in serotonin levels was observed at 2.3 ng/mL compared to the NOR group (Figure 3). The serotonin level of PC was 2.7 ng/mL, showing an increasing trend compared with the LOP group, but there was no statistically significant difference (Figure 3). On the other hand, the serotonin concentration in the G1 groups was 2.5 ng/mL, that of G2 was 4.1 ng/mL, and that of G3 was 4.0 ng/mL, and the low-concentration G1 group did not show significant changes; however, it showed a slight increase (Figure 3). In the G2 and G3 groups, a significant increase in the secretion of serotonin was observed in the colon compared to the LOP group (Figure 3). This result shows that multi-strain probiotics improve constipation by increasing the secretion of serotonin in the colon.

### Metabolites

**Figure 4:**
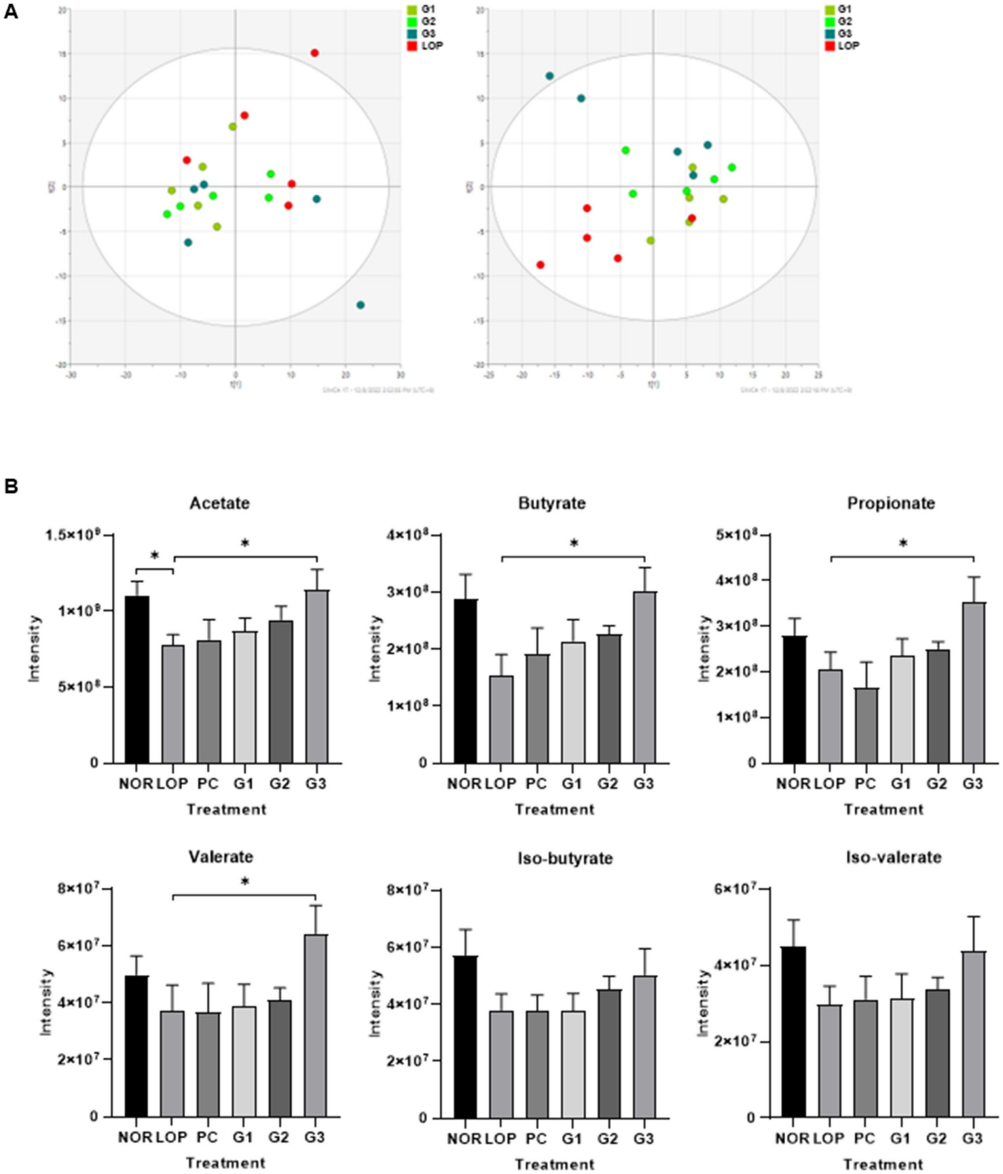
Changes in untargeted metabolites and short-chain fatty acids (SCFAs) by induction of constipation and administration of multi-strain probiotics Consti-Biome. **(A)** left indicate Principal Component Analysis (PCA) and right indicate Partial Least Squares Discriminant Analysis (PLS-DA) of metabolite profiling in fecal of loperamide administration (LOP), low concentration multi-strain probiotics administration (G1, 2 × 10^8^ cfu/mL), medium concentration multi-strain probiotics administration (G2, 2 × 10^9^ cfu/mL), and high concentration probiotic administration (G3, 2 × 10^10^ cfu/mL) groups. **(B)** Intensity of fecal SCFAs in normal control (NOR), LOP, positive control (PC), G1, G2, and G3. Data are mean ± SEM of 5 animals in **(B)**. *p < 0.05, **p < 0.01, significantly different from the LOP as per Mann–whitney *u*-test.

A total of 256 valid metabolites from all samples were identified through LC–MS analysis. As shown in Figure 4A, the predictive ability parameter Q2cum and goodness-of-fit parameter R2X of the first left principal component analysis (PCA) were 0.311 and 0.53, respectively. In PLS-DA, it was observed that each group was distinguished from the LOP group as the concentration of multi-strain probiotics increased according to component 2. PLS-DA score plots discriminated between the constipation induction group and the probiotics treatment group after constipation induction with Q2cum (0.0826), R2X (0.51), and R2Y (0.344).

The common or unique metabolites are further examined that were regulated in the LOP and probiotics treatment groups (G1, G2, and G3 group). A total of common metabolites showed similar decreasing patterns among the three treatment groups compared to LOP group. Furthermore, it was confirmed that SCFAs (Acetate, Propionate, Butyrate, Valerate) increased significantly only in the G3 group, which was fed the highest dose (Supplementary Figure S3; Supplementary Tables S1–S3).

The six short-chain fatty acids (SCFAs) are listed in Figure 4B. Although the differences of SCFAs were not significant among the total 6 groups, acetate was significantly decreased in the LOP group compared to the NOR group, and the G3 group was shown to be able to significantly restore the concentration of acetate that was decreased by the induction of constipation. Butyrate, propionate, and valerate showed dose-dependent increasing trend in all the probiotic intake groups, but a significant change was observed only in the G3 group. Except for the isomers iso-butyrate and iso-valerate, no significant changes were observed among the groups, but their intensity increased with an increase in the concentration of multi-strain probiotics (Figure 4B; Supplementary Table S4).

### Microbiome change

**Figure 5:**
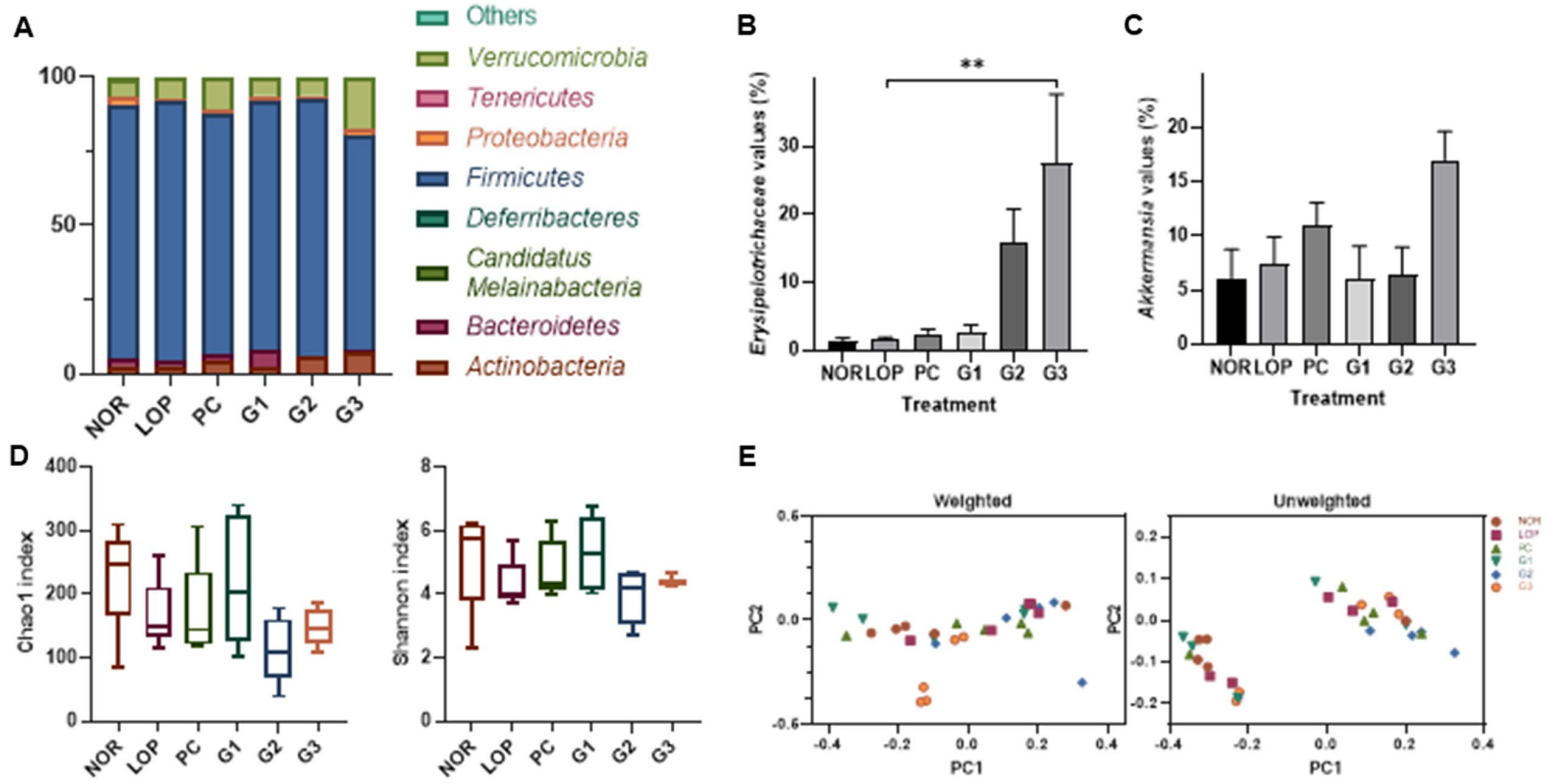
Microbiome changes by induction of constipation and intake of multi-strain probiotics Consti-Biome. **(A)** Is the representative phylum increase and decrease. **(B,C)** Are variations of the family *Erysipelotrichaceae* and the genus *Akkermansia*, respectively. **(D,E)** Exhibit the alpha-diversity beta-diversity, respectively. Microbiome of normal control (NOR), loperamide administration (LOP), positive control (PC), low concentration multi-strain probiotics administration (G1, 2 × 10^8^ cfu/mL), medium concentration multi-strain probiotics administration (G2, 2 × 10^9^ cfu/mL), and high concentration probiotic administration (G3, 2 × 10^10^ cfu/mL) groups were analyzed with fecal samples 2 weeks after treatment with multi-strain probiotics. Data are mean ± SEM of 5 animals in **B** and **C**. *p < 0.05, significantly different from the LOP as per one-way ANOVA with the Tukey’s post-test.

Changes in bacteria of stools were observed in the analysis of the microbiome (Supplementary Figure S5). At the phylum level, *Firmicutes* occupied the highest proportion in all groups (Figure 5A). Followed by the t-test not in ANOVA, the phylum *Verrucomicrobiota* decreased in abundance in the LOP group compared to the NOR group and significantly increased in the G3 group (Figure 5A; Supplementary Figure S4). At the family level, *Erysipelotrichaceae* belonging to the phylum *Firmicutes* (now *Bacillota*) was found to increase in abundance in the G2 and G3 groups compared to the LOP (Figure 5B). The genus *Akkermansia* belonging to *Verrucomicrobiota* increased in the G3 group (Figure 5C; Supplementary Figure S4). However, significant differences were not observed among the groups in alpha diversity and beta diversity (Figures 5D,F). In the PCoA analysis, each group was not clearly separated. In PCA for weighted, the percent variation explained for PC1 was 44.36% and for PC2 was 26.86%. In unweighted, 33.63% by PC1 and 8.51% by PC2 were explained (Figure 5E).

## Discussion

Constipation is one of the most common gastrointestinal (GI) disorders worldwide. To alleviate this, probiotics are often used. In this study, the relieving effect of a mixture of the genera *Lactobacillus, Bifidobacterium, and Streptococcus* on constipation was assessed. Multi-strain probiotics consisted of SynBalance® SmilinGut (*Lactobacillus plantarum, Lactobacillus rhamnosus*, *Bifidobacterium animalis* subsp. *lactis*; ROELMI), *L. plantarum* (UAS), *Lactobacillus acidophilus* (UAS), and *Streptococcus thermophilus* (Chong Kun Dang Bio). The probiotics improved constipation and increased the mRNA expression of genes related to serotonin and mucin in the colon. Cytokine-related genes tend to be decreased in G2 and G3. Increased colonic serotonin production and fecal short-chain fatty acids (SCFAs) were also observed. In fecal samples treated with the probiotics, the abundances of the phylum *Verrucomicrobiota*, the family *Erysipelotrichaceae*, and the genus *Akkermansia* were increased.

Previously, SmilinGut was used in a double-blind clinical trial with 50 subjects. Constipation was evaluated through self-symptom scoring. It was confirmed in the probiotic administration group that more than 30% of the subjects had improvement in their symptoms. It has been found to colonize well in the GI tract (Mezzasalma et al., 2016). However, clear mechanism has not been elucidated. Here, the mixture of the SmilinGut and other probiotics showed an improvement effect on symptoms related to constipation. The probiotic mixture used in this experiment improved constipation indicators such as the stool number, pH, and water content *in vivo* (Figures 1B–D). A high pH and low water content may be due to slow transit in GI. The probiotics also recovered the changes in the GI transit rate induced by loperamide (Figure 1E). Among these symptoms, GI transit time and water content are important risk factors for constipation (Burkitt et al., 1972). Several studies on the improvement in constipation by probiotics have been reported. *B. animalis* subsp. *lactis* exhibited an increase in the GI transit rate in both animal and clinical trials (Wang et al., 2021). Gut motility was increased after *Lactobacillus plantarum* was administered to subjects with constipation (Kusumo et al., 2019). In both of these strains, an increase in SCFAs was confirmed by changes in the gut microbiome. All things considered, the positive effect on constipation by the probiotic mixture used in this study could be due to the improvement in the GI tract.

5-HT (5-hydroxytryptamine; 5-HT) could be altered in disorders associated with changes in bowel function and sensation. Enterochromaffin cells use tryptophan hydroxylase (TPH) to synthesize 5-HT. TPH includes Tph1, which is mainly involved in peripheral serotonin synthesis, and TPH2, which is an isoenzyme involved in serotonin synthesis in the central nervous system, such as the raphe nucleus (Walther et al., 2003). 5-HT is removed from the interstitial space via the serotonin-selective reuptake transporter (Sert). Sert can induce chronic constipation by attenuating intestinal motility and circulatory muscle contractile activity (Guarino et al., 2011). 5-HT is known to decrease in the colonic mucosa of patients with chronic constipation (Coates et al., 2004). Increasing the concentration of 5-HT could decrease the colonic transit time of feces in rats and was associated with SCFAs (Fukumoto et al., 2003). Therefore, 5-HT is one of the important mechanisms for improving constipation. In a constipation rat model induced by loperamide, treatment with *Bifidobacterium bifidum* ameliorated symptoms of constipation, and increases in the 5-HT and Tph1 mRNA levels were observed (Makizaki et al., 2021). Treatment with *Bifidobacterium animalis* subsp. *lactis* in zebrafish could upregulate genes for 5-HT synthesis and downregulate Sert, which was associated with SCFAs (Lu et al., 2019). *B. animalis* was able to increase Muc2 as well as serotonin (Lu et al., 2021). This result was also observed when loperamide-induced constipation mice were treated with *Bifidobacterium lactis* (Tang et al., 2022). Similarly, in this study, an increase in serotonin and mucin in the colon was observed (Figures 2, 3). Muc2 is a mucin that forms a protective layer mucus that covers intestinal epithelial cells and helps maintain the integrity of the intestinal mucosal barrier (Wlodarska et al., 2014). An increase in mucin for constipation treatment has been continuously observed. This can be reduced by constipation (Kim et al., 2019a) and restored by probiotics or substances or a combination of probiotics and other substances (Kim et al., 2013, 2019b; Huang et al., 2022; Tang et al., 2022). Although significant results regarding intestinal mucosal length and thickness were not observed, they showed an increasing tendency (Supplementary Figure S1). In the long term, the results might support an improvement in intestinal mucosal length and thickness.

Constipation is related to impairment in the intestinal mucosal immune system (Collins, 1996). Cytokines are important mediators of inflammation and immune responses and include Tnf-α, Tnf-β, Il-8, and Il-10. These are known to increase in constipated patients (Zhou et al., 2015). *Bifidobacterium longum* reduced serum Tnf-α induced by loperamide in mice (Wang et al., 2022). Similarly, *Lacticaseibacillus paracasei* reduced serum Tnf-α in human experiments (Zhang et al., 2021). Although a significant difference was not observed, as the concentration of the probiotic mixture increased, Tnf-α expression was decreased in the colon of rats (Figure 2). This tendency might help avoid the disruption of tight junction proteins and the consequent increase in intestinal permeability (Zhou et al., 2015).

Alterations in metabolites are known to be caused by disease induction or the consumption of probiotics. *Lactobacillus* spp. and *Bifidobacterium* spp. can produce local metabolites (Wilms et al., 2021). Here, *in vivo*, changes in the metabolites in fecal samples by the mixture of probiotics that included *Lactobacillus* and *Bifidobacterium* were observed. Metabolites were clearly distinguished in partial squares discriminant analysis depending on the increasing concentration of probiotics (Figure 5A). A significant increase in SCFAs was observed, which are known to have positive effects on various diseases (Figure 4B). SCFAs are one of the key mechanisms by which probiotics improve chronic constipation. Among SCFAs, acetate, butyrate, propionate, and valerate were increased in rat cecal contents (Figure 4B). Treatment with probiotics for constipation has shown similar results (Wang et al., 2017; Zhao et al., 2022). In animal experiments, the absorption issue of SCFAs in the small intestine was resolved when acylated starches were fed for 1 month (Wang et al., 2020). These starches ameliorated constipation by increasing acetate-producing bacteria, which had a positive correlation with the small intestinal transit rate and water content of fecal samples (Wang et al., 2020). On the other hand, in the group consuming butylated starch, constipation was alleviated by butyrate-producing bacteria, which had a negative correlation with the first black stool defecation time (Wang et al., 2020). Although propionate did not directly relieve constipation, it was consistently observed in the feces of experimental groups fed probiotics in the constipation group (Zhuang et al., 2019; Wang et al., 2020, 2022). Its constipation-inhibiting effect might be due to an interaction control mechanism with butyrate (Zhuang et al., 2019). Valerate is also known to correlate with other SCFAs and the microbiome (Lu et al., 2022).

In addition, various constipation relief mechanisms of the SCFAs of probiotics have been studied. SCFAs can decrease luminal pH and improve gut motility by stimulating the contraction of colonic smooth muscle (Wilms et al., 2021). The activation of goblet cells and the increase in mucin-related gene expression are also closely related to SCFAs (Caballero-Franco et al., 2007). SCFAs have a local effect in the gut and affect intestinal immune cells (Ratajczak et al., 2019). In addition, they are also involved in colonic serotonin production (Reigstad et al., 2015). Therefore, SCFAs produced by probiotics could not only improve the pH and physical and chemical properties of the GI tract but could also stimulate the production of serotonin and mucin. These factors might contribute to constipation amelioration.

Numerous studies have shown that the microbiome affects host health through various mechanisms. It was observed that the phylum *Verrucomicrobiota*, the family *Erysipelotrichaceae*, and genus *Akkermansia* were significantly increased compared to the control group in this study (Figures 5A–C). An increase in *Erysipelotrichaceae* was observed in an animal model in which constipation was alleviated by Raffino-Oligosaccharide treatment (Liang et al., 2022). *Erysipelotrichaceae* are known to produce butyrate, one of the SCFAs. Butyrate can maintain barrier integrity by accelerating GI transit (Soret et al., 2010; Zhuang et al., 2019).

The genus *Akkermansia*, which was increased in this experiment (Figure 5C), was recently proposed as a next-generation probiotic (Liu Y. et al., 2022). The *Akkermansia* genus belongs to the phylum *Verrucomicrobiota*. Increasing levels of *Akkermansia* might significantly contribute to the increase in *Verrucomicrobiota* abundance identified by microbiome analysis here. Two species of *Akkermansia*, namely, *Akkermansia glycaniphila* and *Akkermansia muciniphila*, were reported at the time of writing (www.bacterio.net/index.html; Parte, 2018). Among them, studies on *A. muciniphila* have been relatively numerous. Mucosal health and mucosal immunity are proposed as the main potential mechanisms affecting host intestinal diseases (Liu M. J. et al., 2022). *A. muciniphila* is a commensal bacterium that could contribute to the maintenance of the integrity (Reunanen et al., 2015) and dynamics (Kim et al., 2014; Singanayagam et al., 2022) of intestinal mucus. This bacterium is observed to decrease with the occurrence of various diseases and to increase with disease relief. An increase in *Akkermansia* abundance was observed following alleviation of irritable bowel syndrome (IBS) and inflammatory bowel disease (IBD) (Png et al., 2010; Rajilić-Stojanović et al., 2013). In animal studies, *A. muciniphila* and its extracellular vesicles alleviated colitis induced by dextran sulfate sodium (Kang et al., 2013; Zhai et al., 2019). *A. muciniphila* could reduce the expression of proinflammatory cytokines such as Tnf-α and IFN-γ in the colon (Zhai et al., 2019). Its outer membrane protein, Amuc-1,100 (Cani and Knauf, 2021), enhances intestinal barrier protection by activating Toll-like receptor 2 (TLR2)-mediated intracellular signaling in intestinal epithelial cells (Plovier et al., 2017). Furthermore, *Akkermansia* contributes to reducing the risk of metabolic syndrome, thereby alleviating complications of obesity and diabetes (Hagi and Belzer, 2021; Hasani et al., 2021; Yoon et al., 2021; Zhou et al., 2021). In addition, it can produce SCFAs (Swidsinski et al., 2009; Png et al., 2010; Arumugam et al., 2011; Rajilić-Stojanović et al., 2013). This suggests that the control of intestinal microflora by probiotics can help alleviate constipation.

The multi-strain probiotics used in this study showed improvement compared to the negative control group regardless of concentration in the number of fecal pellets, pH, and water content (Figures 1B–D). In addition, GI transit rate, one of the indicators related to constipation, showed a significant change at concentrations above G2 (Figure 1E). Based on the analyzed mechanisms for improving constipation, 5-HT1a and 5-HT1b, both serotonin-related factors, were significantly expressed in G1 and G2. Although the expression of these mRNA showed a tendency to increase in G3, no significant difference was observed compared to the LOP (Figure 2). However, the concentration of serotonin confirmed at the protein level did not increase in G1. On the other hand, an increase was observed in G3, which was not statistically significant at mRNA levels. Increased protein levels of serotonin were also observed in G2 (Figure 3). The difference between mRNA expression and protein expression may be caused by various factors. The relationship between mRNA levels and proteins can be influenced by steady state, long-term state, and short-term adaptation. Local availability of resources for protein biosynthesis and spatial and temporal mRNA variations also affect the relationship between protein levels and their coding transcripts. In addition, post-transcriptional processes in highly dynamic phases such as cell differentiation or stress response can lead to variation (Liu et al., 2016). Various factors can cause differences in protein and mRNA expression. In addition, supernatants of *Erysipelotrichaceae*, which were significantly observed in G2 and G3, were related to the serotonin pathway (Luna et al., 2017). Similarly, *Akkermansia* is associated with SCFAs (Koh et al., 2016). Therefore, microbiome changes may be closely involved in the expression of mRNAs, proteins, and SCFAs.

The multi-strain probiotics used in this study could modulate the microbiome and SCFAs and increase serotonin, mucin, and the GI transit rate. In addition, the number, pH, and water content in stools were improved. This treatment could relieve the symptoms of constipation. Taken together, the findings show that the bacterial mixture might increase the secretion of SCFAs, and as a result, constipation could be improved by increasing serotonin and mucin. Therefore, practically, this mixture could be applied to patients with constipation.

## Conclusion

A multi-strain probiotics Consti-Biome containing a mixture of SynBalance® SmilinGut (*Lactobacillus plantarum, Lactobacillus rhamnosus*, *Bifidobacterium animalis* subsp. *lactis*; ROELMI), *L. plantarum* (UAS), *Lactobacillus acidophilus* (UAS), and *Streptococcus thermophilus* (Chong Kun Dang Bio) improved the number of fecal pellets, pH, and water content and gastrointestinal (GI) transit rate in rats with constipation induced by loperamide. The multi-strain probiotics could also increase the expression levels of serotonin and mucin-related genes. The improvement in constipation by the multi-strain probiotics could be a result of increasing the production of short-chain fatty acids (SCFAs) by altering the microbial composition, including the genus *Akkermansia.* These results showed the positive potential of using the probiotics in the treatment of constipation. However, clinical trials will be needed to evaluate the efficacy and effectiveness of the probiotics.

## Data availability statement

The original contributions presented in the study are included in the article/Supplementary material, further inquiries can be directed to the corresponding authors.

## Ethics statement

The animal study was reviewed and approved by Animal Experimental Ethics Committee (IACUC) of Hallym University (Hallym 2021-79).

## Author contributions

B-YK and KTS contributed to conception and design of the study. J-JJ, RG, Y-JJ, HJP, BHM, MKJ, SJY, MRC, JC, and JHM organized the database and performed the statistical analysis. J-JJ wrote the first draft of the manuscript. RG, Y-JJ, MRC wrote sections of the manuscript. All authors contributed to manuscript revision, read, and approved the submitted version.

## Funding

This research was supported by Hallym University Research Fund, the Basic Science Research Program through the National Research Foundation of Korea (NRF) funded by the Ministry of Education, Science and Technology (NRF-2020R1A6A1A03043026 and 2020R1I1A3073530), Korea Institute for Advancement of Technology (P0020622), and Bio Industrial Technology Development Program (20018494) funded by the Ministry of Trade, Industry and Energy (MOTIE, Korea).

## Conflict of interest

The authors declare that the research was conducted in the absence of any commercial or financial relationships that could be construed as a potential conflict of interest.

## Publisher’s note

All claims expressed in this article are solely those of the authors and do not necessarily represent those of their affiliated organizations, or those of the publisher, the editors and the reviewers. Any product that may be evaluated in this article, or claim that may be made by its manufacturer, is not guaranteed or endorsed by the publisher.

## Supplementary material

The Supplementary material for this article can be found online at: https://www.frontiersin.org/articles/10.3389/fmicb.2023.1174968/full#supplementary-material

## Abbreviations

GI: Gastrointestinal
SCFAs: Short-chain fatty acids
D-PBS: Dulbecco’s phosphate-buffered saline

## References

[ref1] Ambizas E. M. Ginzburg R. (2007). Lubiprostone: a chloride channel activator for treatment of chronic constipation. Ann. Pharmacother. 41, 957–964. doi: 10.1345/aph.1K047 17519292

[ref2] Ao M. Sarathy J. Domingue J. Alrefai W. A. Rao M. C. (2013). Chenodeoxycholic acid stimulates cl–secretion via cAMP signaling and increases cystic fibrosis transmembrane conductance regulator phosphorylation in T84 cells. Am. J. Phys. Cell Phys. 305, C447–C456. doi: 10.1152/ajpcell.00416.2012, PMID: 23761628 PMC3891218

[ref3] Arora T. Sharma R. (2011). Fermentation potential of the gut microbiome: implications for energy homeostasis and weight management. Nutr. Rev. 69, 99–106. doi: 10.1111/j.1753-4887.2010.00365.x, PMID: 21294743

[ref4] Arumugam M. Raes J. Pelletier E. Le Paslier D. Yamada T. Mende D. R.. (2011). Enterotypes of the human gut microbiome. Nature 473, 174–180. doi: 10.1038/nature09944, PMID: 21508958 PMC3728647

[ref5] Böcker U. Nebe T. Herweck F. Holt L. Panja A. Jobin C.. (2003). Butyrate modulates intestinal epithelial cell-mediated neutrophil migration. Clin. Exp. Immunol. 131, 53–60. doi: 10.1046/j.1365-2249.2003.02056.x, PMID: 12519386 PMC1808611

[ref6] Bosaeus I. (2004). Fibre effects on intestinal functions (diarrhoea, constipation and irritable bowel syndrome). Clin. Nutr. Suppl. 1, 33–38. doi: 10.1016/j.clnu.2004.09.006

[ref7] Burkitt D. P. Walker A. Painter N. S. (1972). Effect of dietary fibre on stools and transit-times, and its role in the causation of disease. Lancet 300, 1408–1411. doi: 10.1016/S0140-6736(72)92974-1, PMID: 4118696

[ref8] Caballero-Franco C. Keller K. De Simone C. Chadee K. (2007). The VSL# 3 probiotic formula induces mucin gene expression and secretion in colonic epithelial cells. Am. J. Physiol. Gastrointest. Liver Physiol. 292, G315–G322. doi: 10.1152/ajpgi.00265.2006 16973917

[ref9] Callahan B. J. Mcmurdie P. J. Rosen M. J. Han A. W. Johnson A. J. A. Holmes S. P. (2016). DADA2: high-resolution sample inference from Illumina amplicon data. Nat. Methods 13, 581–583. doi: 10.1038/nmeth.3869, PMID: 27214047 PMC4927377

[ref10] Camilleri M. (2009). Serotonin in the gastrointestinal tract. Curr. Opin. Endocrinol. Diabetes Obes. 16, 53–59. doi: 10.1097/MED.0b013e32831e9c8e, PMID: 19115522 PMC2694720

[ref11] Cani P. D. Knauf C. (2021). A newly identified protein from *Akkermansia muciniphila* stimulates GLP-1 secretion. Cell Metab. 33, 1073–1075. doi: 10.1016/j.cmet.2021.05.004, PMID: 34077715

[ref12] Cao H. Liu X. An Y. Zhou G. Liu Y. Xu M.. (2017). Dysbiosis contributes to chronic constipation development via regulation of serotonin transporter in the intestine. Sci. Rep. 7:10322. doi: 10.1038/s41598-017-10835-8, PMID: 28871143 PMC5583244

[ref13] Caporaso J. G. Kuczynski J. Stombaugh J. Bittinger K. Bushman F. D. Costello E. K.. (2010). QIIME allows analysis of high-throughput community sequencing data. Nat. Methods 7, 335–336. doi: 10.1038/nmeth.f.303, PMID: 20383131 PMC3156573

[ref14] Chen H.-J. Dai F.-J. Chang C.-R. Lau Y.-Q. Chew B.-S. Chau C.-F. (2019). Impact of dietary ingredients on the interpretation of various fecal parameters in rats fed inulin. J. Food Drug Anal. 27, 869–875. doi: 10.1016/j.jfda.2019.06.005, PMID: 31590758 PMC9306980

[ref15] Coates M. D. Mahoney C. R. Linden D. R. Sampson J. E. Chen J. Blaszyk H.. (2004). Molecular defects in mucosal serotonin content and decreased serotonin reuptake transporter in ulcerative colitis and irritable bowel syndrome. Gastroenterology 126, 1657–1664. doi: 10.1053/j.gastro.2004.03.013 15188158

[ref16] Collins S. M. (1996). The immunomodulation of enteric neuromuscular function: implications for motility and inflammatory disorders. Gastroenterology 111, 1683–1699. doi: 10.1016/S0016-5085(96)70034-3, PMID: 8942751

[ref17] Costedio M. M. Hyman N. Mawe G. M. (2007). Serotonin and its role in colonic function and in gastrointestinal disorders. Dis. Colon Rectum 50, 376–388. doi: 10.1007/s10350-006-0763-3 17195902

[ref18] Dimidi E. Scott S. M. Whelan K. (2020). Probiotics and constipation: mechanisms of action, evidence for effectiveness and utilisation by patients and healthcare professionals. Proc. Nutr. Soc. 79, 147–157. doi: 10.1017/S0029665119000934, PMID: 31262376

[ref19] Eor J. Y. Tan P. L. Lim S. M. Choi D. H. Yoon S. M. Yang S. Y.. (2019). Laxative effect of probiotic chocolate on loperamide-induced constipation in rats. Food Res. Int. 116, 1173–1182. doi: 10.1016/j.foodres.2018.09.062, PMID: 30716903

[ref20] Faigel D. O. (2002). A clinical approach to constipation. Clin. Cornerstone 4, 11–18. doi: 10.1016/S1098-3597(02)90002-5 12739323

[ref21] Forootan M. Bagheri N. Darvishi M. (2018). Chronic constipation: a review of literature. Medicine 97:e10631. doi: 10.1097/MD.0000000000010631, PMID: 29768326 PMC5976340

[ref22] Fukuda S. Toh H. Hase K. Oshima K. Nakanishi Y. Yoshimura K.. (2011). Bifidobacteria can protect from enteropathogenic infection through production of acetate. Nature 469, 543–547. doi: 10.1038/nature09646, PMID: 21270894

[ref23] Fukumoto S. Tatewaki M. Yamada T. Fujimiya M. Mantyh C. Voss M.. (2003). Short-chain fatty acids stimulate colonic transit via intraluminal 5-HT release in rats. Am. J. Phys. Regul. Integr. Comp. Phys. 284, R1269–R1276. doi: 10.1152/ajpregu.00442.2002 12676748

[ref24] Gershon M. (2004). Serotonin receptors and transporters—roles in normal and abnormal gastrointestinal motility. Aliment. Pharmacol. Ther. 20, 3–14. doi: 10.1111/j.1365-2036.2004.02180.x, PMID: 15521849

[ref25] Guarino M. Cheng L. Cicala M. Ripetti V. Biancani P. Behar J. (2011). Progesterone receptors and serotonin levels in colon epithelial cells from females with slow transit constipation. Neurogastroenterol. Motil. 23, 575–e210. doi: 10.1111/j.1365-2982.2011.01705.x, PMID: 21481100

[ref26] Hagi T. Belzer C. (2021). The interaction of *Akkermansia muciniphila* with host-derived substances, bacteria and diets. Appl. Microbiol. Biotechnol. 105, 4833–4841. doi: 10.1007/s00253-021-11362-3, PMID: 34125276 PMC8236039

[ref27] Han J. Lin K. Sequeira C. Borchers C. H. (2015). An isotope-labeled chemical derivatization method for the quantitation of short-chain fatty acids in human feces by liquid chromatography–tandem mass spectrometry. Anal. Chim. Acta 854, 86–94. doi: 10.1016/j.aca.2014.11.015, PMID: 25479871

[ref28] Hasani A. Ebrahimzadeh S. Hemmati F. Khabbaz A. Hasani A. Gholizadeh P. (2021). The role of *Akkermansia muciniphila* in obesity, diabetes and atherosclerosis. J. Med. Microbiol. 70:001435. doi: 10.1099/jmm.0.001435 34623232

[ref29] Hatayama H. Iwashita J. Kuwajima A. Abe T. (2007). The short chain fatty acid, butyrate, stimulates MUC2 mucin production in the human colon cancer cell line, LS174T. Biochem. Biophys. Res. Commun. 356, 599–603. doi: 10.1016/j.bbrc.2007.03.025, PMID: 17374366

[ref30] Hays S. Jacquot A. Gauthier H. Kempf C. Beissel A. Pidoux O.. (2016). Probiotics and growth in preterm infants: a randomized controlled trial, PREMAPRO study. Clin. Nutr. 35, 802–811. doi: 10.1016/j.clnu.2015.06.006, PMID: 26220763

[ref31] Henningsson Ç. M. Margareta E. Nyman G. Björck I. M. (2001). Content of short-chain fatty acids in the hindgut of rats fed processed bean (*Phaseolus vulgaris*) flours varying in distribution and content of indigestible carbohydrates. Br. J. Nutr. 86, 379–389. doi: 10.1079/BJN2001423, PMID: 11570990

[ref32] Huang J. Lin B. Zhang Y. Xie Z. Zheng Y. Wang Q.. (2022). Bamboo shavings derived O-acetylated xylan alleviates loperamide-induced constipation in mice. Carbohydr. Polym. 276:118761. doi: 10.1016/j.carbpol.2021.118761, PMID: 34823784

[ref33] Jahng J. Jung I. Choi E. Conklin J. Park H. (2012). The effects of methane and hydrogen gases produced by enteric bacteria on ileal motility and colonic transit time. Neurogastroenterol. Motil. 24, 185–e92. doi: 10.1111/j.1365-2982.2011.01819.x, PMID: 22097886

[ref34] Jeong J.-J. Park H. J. Cha M. G. Park E. Won S.-M. Ganesan R.. (2022). The Lactobacillus as a probiotic: focusing on liver diseases. Microorganisms 10:288. doi: 10.3390/microorganisms10020288, PMID: 35208742 PMC8879051

[ref35] Kalina U. Koyama N. Hosoda T. Nuernberger H. Sato K. Hoelzer D.. (2002). Enhanced production of IL-18 in butyrate-treated intestinal epithelium by stimulation of the proximal promoter region. Eur. J. Immunol. 32, 2635–2643. doi: 10.1002/1521-4141(200209)32:9<2635::AID-IMMU2635>3.0.CO;2-N, PMID: 12207348

[ref36] Kang C.-S. Ban M. Choi E.-J. Moon H.-G. Jeon J.-S. Kim D.-K.. (2013). Extracellular vesicles derived from gut microbiota, especially *Akkermansia muciniphila*, protect the progression of dextran sulfate sodium-induced colitis. PLoS One 8:e76520. doi: 10.1371/journal.pone.0076520, PMID: 24204633 PMC3811976

[ref37] Katoh K. Standley D. M. (2013). MAFFT multiple sequence alignment software version 7: improvements in performance and usability. Mol. Biol. Evol. 30, 772–780. doi: 10.1093/molbev/mst010, PMID: 23329690 PMC3603318

[ref38] Kim B.-S. Song M.-Y. Kim H. (2014). The anti-obesity effect of *Ephedra sinica* through modulation of gut microbiota in obese Korean women. J. Ethnopharmacol. 152, 532–539. doi: 10.1016/j.jep.2014.01.038, PMID: 24556223

[ref39] Kim J. E. Lee M. R. Park J. J. Choi J. Y. Song B. R. Son H. J.. (2018). Quercetin promotes gastrointestinal motility and mucin secretion in loperamide-induced constipation of SD rats through regulation of the mAChRs downstream signal. Pharm. Biol. 56, 309–317. doi: 10.1080/13880209.2018.1474932, PMID: 29952685 PMC6130520

[ref40] Kim J. E. Lee Y. J. Kwak M. H. Ko J. Hong J. T. Hwang D. Y. (2013). Aqueous extracts of Liriope platyphylla induced significant laxative effects on loperamide-induced constipation of SD rats. BMC Complement. Altern. Med. 13, 1–12. doi: 10.1186/1472-6882-13-333 24274470 PMC4222752

[ref41] Kim J. E. Park J. W. Kang M. J. Choi H. J. Bae S. J. Choi Y. S.. (2019a). Anti-inflammatory response and muscarinic cholinergic regulation during the laxative effect of *Asparagus cochinchinensis* in loperamide-induced constipation of SD rats. Int. J. Mol. Sci. 20:946. doi: 10.3390/ijms20040946, PMID: 30795644 PMC6412595

[ref42] Kim J. E. Yun W. B. Lee M. L. Choi J. Y. Park J. J. Kim H. R.. (2019b). Synergic laxative effects of an herbal mixture of *Liriope platyphylla*, *Glycyrrhiza uralensis*, and *Cinnamomum cassia* in loperamide-induced constipation of Sprague Dawley rats. J. Med. Food 22, 294–304. doi: 10.1089/jmf.2018.4234, PMID: 30724689

[ref43] Koebnick C. Wagner I. Leitzmann P. Stern U. Zunft H. (2003). Probiotic beverage containing *Lactobacillus casei* Shirota improves gastrointestinal symptoms in patients with chronic constipation. Can. J. Gastroenterol. 17, 655–659. doi: 10.1155/2003/654907, PMID: 14631461

[ref44] Koh A. De Vadder F. Kovatcheva-Datchary P. Bäckhed F. (2016). From dietary fiber to host physiology: short-chain fatty acids as key bacterial metabolites. Cells 165, 1332–1345. doi: 10.1016/j.cell.2016.05.041, PMID: 27259147

[ref45] Kusumo P. D. Maulahela H. Utari A. P. Surono I. S. Soebandrio A. Abdullah M. (2019). Probiotic *Lactobacillus plantarum* IS 10506 supplementation increase SCFA of women with functional constipation. Iran. J. Microbiol. 11, 389–396. doi: 10.18502/ijm.v11i5.1957 PMID: 32148669 PMC7049320

[ref46] Kwoji I. D. Aiyegoro O. A. Okpeku M. Adeleke M. A. (2021). Multi-strain probiotics: synergy among isolates enhances biological activities. Biology 10:322. doi: 10.3390/biology10040322, PMID: 33924344 PMC8070017

[ref47] Li C. Nie S.-P. Zhu K.-X. Xiong T. Li C. Gong J.. (2015). Effect of *Lactobacillus plantarum* NCU116 on loperamide-induced constipation in mice. Int. J. Food Sci. Nutr. 66, 533–538. doi: 10.3109/09637486.2015.1024204, PMID: 25822005

[ref48] Liang Y. Wang Y. Wen P. Chen Y. Ouyang D. Wang D.. (2022). The anti-constipation effects of raffino-oligosaccharide on gut function in mice using neurotransmitter analyses, 16S rRNA sequencing and targeted screening. Molecules 27:2235. doi: 10.3390/molecules27072235, PMID: 35408632 PMC9000249

[ref49] Liu M.-J. Yang J.-Y. Yan Z.-H. Hu S. Li J.-Q. Xu Z.-X.. (2022). Recent findings in *Akkermansia muciniphila*-regulated metabolism and its role in intestinal diseases. Clin. Nutr. 41, 2333–2344. doi: 10.1016/j.clnu.2022.08.029, PMID: 36113229

[ref50] Liu Y. Beyer A. Aebersold R. (2016). On the dependency of cellular protein levels on mRNA abundance. Cells 165, 535–550. doi: 10.1016/j.cell.2016.03.014 27104977

[ref51] Liu Y. Yang M. Tang L. Wang F. Huang S. Liu S.. (2022). TLR4 regulates RORγt+ regulatory T-cell responses and susceptibility to colon inflammation through interaction with *Akkermansia muciniphila*. Microbiome 10, 1–20. doi: 10.1186/s40168-022-01296-x 35761415 PMC9235089

[ref52] Lu D. Pi Y. Ye H. Wu Y. Bai Y. Lian S.. (2022). Consumption of dietary Fiber with different physicochemical properties during late pregnancy alters the gut microbiota and relieves constipation in sow model. Nutrients 14:2511. doi: 10.3390/nu14122511, PMID: 35745241 PMC9229973

[ref53] Lu Y. Yu Z. Zhang Z. Liang X. Gong P. Yi H.. (2021). *Bifidobacterium animalis* F1-7 in combination with konjac glucomannan improves constipation in mice via humoral transport. Food Funct. 12, 791–801. doi: 10.1039/D0FO02227F, PMID: 33393951

[ref54] Lu Y. Zhang Z. Liang X. Chen Y. Zhang J. Yi H.. (2019). Study of gastrointestinal tract viability and motility via modulation of serotonin in a zebrafish model by probiotics. Food Funct. 10, 7416–7425. doi: 10.1039/C9FO02129A, PMID: 31660551

[ref55] Luna R. A. Oezguen N. Balderas M. Venkatachalam A. Runge J. K. Versalovic J.. (2017). Distinct microbiome-neuroimmune signatures correlate with functional abdominal pain in children with autism spectrum disorder. Cell. Mol. Gastroenterol. Hepatol. 3, 218–230. doi: 10.1016/j.jcmgh.2016.11.008, PMID: 28275689 PMC5331780

[ref56] Makizaki Y. Uemoto T. Yokota H. Yamamoto M. Tanaka Y. Ohno H. (2021). Improvement of loperamide-induced slow transit constipation by *Bifidobacterium bifidum* G9-1 is mediated by the correction of butyrate production and neurotransmitter profile due to improvement in dysbiosis. PLoS One 16:e0248584. doi: 10.1371/journal.pone.0248584, PMID: 33750988 PMC7984621

[ref57] Martin M. (2011). Cutadapt removes adapter sequences from high-throughput sequencing reads. EMBnet. J. 17, 10–12. doi: 10.14806/ej.17.1.200

[ref58] Mezzasalma V. Manfrini E. Ferri E. Sandionigi A. La Ferla B. Schiano I.. (2016). A randomized, double-blind, placebo-controlled trial: the efficacy of multispecies probiotic supplementation in alleviating symptoms of irritable bowel syndrome associated with constipation. Biomed. Res. Int. 2016:4740907. doi: 10.1155/2016/4740907 27595104 PMC4993960

[ref59] Myllyluoma E. Veijola L. Ahlroos T. Tynkkynen S. Kankuri E. Vapaatalo H.. (2005). Probiotic supplementation improves tolerance to *Helicobacter pylori* eradication therapy–a placebo-controlled, double-blind randomized pilot study. Aliment. Pharmacol. Ther. 21, 1263–1272. doi: 10.1111/j.1365-2036.2005.02448.x, PMID: 15882248

[ref60] Park M. I. (2011). Treatment of constipation. Korean J. Med. 80, 510–523.

[ref61] Parte A. C. (2018). LPSN–list of prokaryotic names with standing in nomenclature (Bacterio. Net), 20 years on. Int. J. Syst. Evol. Microbiol. 68, 1825–1829. doi: 10.1099/ijsem.0.002786, PMID: 29724269

[ref62] Plaza-Diaz J. Ruiz-Ojeda F. J. Gil-Campos M. Gil A. (2019). Mechanisms of action of probiotics. Adv. Nutr. 10, S49–S66. doi: 10.1093/advances/nmy063, PMID: 30721959 PMC6363529

[ref63] Plovier H. Everard A. Druart C. Depommier C. Van Hul M. Geurts L.. (2017). A purified membrane protein from *Akkermansia muciniphila* or the pasteurized bacterium improves metabolism in obese and diabetic mice. Nat. Med. 23, 107–113. doi: 10.1038/nm.4236, PMID: 27892954

[ref64] Png C. W. Lindén S. K. Gilshenan K. S. Zoetendal E. G. Mcsweeney C. S. Sly L. I.. (2010). Mucolytic bacteria with increased prevalence in IBD mucosa augmentin vitroutilization of mucin by other bacteria. Am. J. Gastroenterol. 105, 2420–2428. doi: 10.1038/ajg.2010.281, PMID: 20648002

[ref65] Rajilić-Stojanović M. Shanahan F. Guarner F. De Vos W. M. (2013). Phylogenetic analysis of dysbiosis in ulcerative colitis during remission. Inflamm. Bowel Dis. 19, 481–488. doi: 10.1097/MIB.0b013e31827fec6d, PMID: 23385241

[ref66] Rambaut A. (2009). *FigTree* v1.3.1. Available at: http://tree.bio.ed.ac.uk/software/figtree/.

[ref67] Ratajczak W. Rył A. Mizerski A. Walczakiewicz K. Sipak O. Laszczyńska M. (2019). Immunomodulatory potential of gut microbiome-derived short-chain fatty acids (SCFAs). Acta Biochim. Pol. 66, 1–12. doi: 10.18388/abp.2018_2648, PMID: 30831575

[ref68] Reigstad C. S. Salmonson C. E. Iii J. F. R. Szurszewski J. H. Linden D. R. Sonnenburg J. L.. (2015). Gut microbes promote colonic serotonin production through an effect of short-chain fatty acids on enterochromaffin cells. FASEB J. 29, 1395–1403. doi: 10.1096/fj.14-259598, PMID: 25550456 PMC4396604

[ref69] Reunanen J. Kainulainen V. Huuskonen L. Ottman N. Belzer C. Huhtinen H.. (2015). *Akkermansia muciniphila* adheres to enterocytes and strengthens the integrity of the epithelial cell layer. Appl. Environ. Microbiol. 81, 3655–3662. doi: 10.1128/AEM.04050-14, PMID: 25795669 PMC4421065

[ref70] Ríos-Covián D. Ruas-Madiedo P. Margolles A. Gueimonde M. De Los Reyes-Gavilán C. G. Salazar N. (2016). Intestinal short chain fatty acids and their link with diet and human health. Front. Microbiol. 7:185. doi: 10.3389/fmicb.2016.00185 26925050 PMC4756104

[ref71] Sin H.-J. Kim K.-O. Kim S.-H. Kim Y.-A. Lee H.-S. (2010). Effect of resistant starch on the large bowel environment and plasma lipid in rats with loperamide-induced constipation. J. Korean Soc. Food Sci. Nutr. 39, 684–691. doi: 10.3746/jkfn.2010.39.5.684

[ref72] Singanayagam A. Footitt J. Marczynski M. Radicioni G. Cross M. T. Finney L. J.. (2022). Airway mucins promote immunopathology in virus-exacerbated chronic obstructive pulmonary disease. J. Clin. Invest. 132:e120901. doi: 10.1172/JCI120901, PMID: 35239513 PMC9012283

[ref73] Singh N. Gurav A. Sivaprakasam S. Brady E. Padia R. Shi H.. (2014). Activation of Gpr109a, receptor for niacin and the commensal metabolite butyrate, suppresses colonic inflammation and carcinogenesis. Immunity 40, 128–139. doi: 10.1016/j.immuni.2013.12.007, PMID: 24412617 PMC4305274

[ref74] Soret R. Chevalier J. De Coppet P. Poupeau G. Derkinderen P. Segain J. P.. (2010). Short-chain fatty acids regulate the enteric neurons and control gastrointestinal motility in rats. Gastroenterology 138:e1774, 1772–1782.e4. doi: 10.1053/j.gastro.2010.01.053 20152836

[ref75] Swidsinski A. Loening-Baucke V. Herber A. (2009). Mucosal flora in Crohn's disease and ulcerative colitis-an overview. J. Physiol. Pharmacol. 60, 61–71. PMID: 20224153

[ref76] Tang T. Wang J. Jiang Y. Zhu X. Zhang Z. Wang Y.. (2022). *Bifidobacterium lactis* TY-S01 prevents Loperamide-induced constipation by modulating gut microbiota and its metabolites in mice. Frontiers. Nutrition 9:890314. doi: 10.3389/fnut.2022.890314 PMC9277448 35845767

[ref77] Walther D. J. Peter J.-U. Bashammakh S. Hortnagl H. Voits M. Fink H.. (2003). Synthesis of serotonin by a second tryptophan hydroxylase isoform. Science 299:76. doi: 10.1126/science.1078197 12511643

[ref78] Wang L. Cen S. Wang G. Lee Y.-K. Zhao J. Zhang H.. (2020). Acetic acid and butyric acid released in large intestine play different roles in the alleviation of constipation. J. Funct. Foods 69:103953. doi: 10.1016/j.jff.2020.103953

[ref79] Wang L. Chai M. Wang J. Qiangqing Y. Wang G. Zhang H.. (2022). *Bifidobacterium longum* relieves constipation by regulating the intestinal barrier of mice. Food Funct. 13, 5037–5049. doi: 10.1039/D1FO04151G, PMID: 35394000

[ref80] Wang L. Chen C. Cui S. Lee Y.-K. Wang G. Zhao J.. (2019). Adhesive Bifidobacterium induced changes in cecal microbiome alleviated constipation in mice. Front. Microbiol. 10:1721. doi: 10.3389/fmicb.2019.01721, PMID: 31456752 PMC6700325

[ref81] Wang L. Hu L. Xu Q. Yin B. Fang D. Wang G.. (2017). *Bifidobacterium adolescentis* exerts strain-specific effects on constipation induced by loperamide in BALB/c mice. Int. J. Mol. Sci. 18:318. doi: 10.3390/ijms18020318, PMID: 28230723 PMC5343854

[ref82] Wang R. Sun J. Li G. Zhang M. Niu T. Kang X.. (2021). Effect of *Bifidobacterium animalis* subsp. *lactis* MN-gup on constipation and the composition of gut microbiota. Benefic. Microbes 12, 31–42. doi: 10.3920/BM2020.0023, PMID: 33308038

[ref83] Wang Y. Guo Y. Chen H. Wei H. Wan C. (2018). Potential of *Lactobacillus plantarum* ZDY2013 and *Bifidobacterium bifidum* WBIN03 in relieving colitis by gut microbiota, immune, and anti-oxidative stress. Can. J. Microbiol. 64, 327–337. doi: 10.1139/cjm-2017-0716, PMID: 29401402

[ref84] Wilms E. An R. Smolinska A. Stevens Y. Weseler A. R. Elizalde M.. (2021). Galacto-oligosaccharides supplementation in prefrail older and healthy adults increased faecal bifidobacteria, but did not impact immune function and oxidative stress. Clin. Nutr. 40, 3019–3031. doi: 10.1016/j.clnu.2020.12.034, PMID: 33509667

[ref85] Wlodarska M. Thaiss C. A. Nowarski R. Henao-Mejia J. Zhang J.-P. Brown E. M.. (2014). NLRP6 inflammasome orchestrates the colonic host-microbial interface by regulating goblet cell mucus secretion. Cells 156, 1045–1059. doi: 10.1016/j.cell.2014.01.026, PMID: 24581500 PMC4017640

[ref86] Yang Y.-X. He M. Hu G. Wei J. Pages P. Yang X.-H.. (2008). Effect of a fermented milk containing *Bifidobacterium lactis* DN-173010 on Chinese constipated women. World J Gastroenterol: WJG 14, 6237–6243. doi: 10.3748/wjg.14.6237, PMID: 18985817 PMC2761588

[ref87] Ye Y. Wang Y. Yang Y. Tao L. (2020). Aloperine suppresses LPS-induced macrophage activation through inhibiting the TLR4/NF-κB pathway. Inflamm. Res. 69, 375–383. doi: 10.1007/s00011-019-01313-0, PMID: 32144444

[ref88] Yoon H. S. Cho C. H. Yun M. S. Jang S. J. You H. J. Kim J.-H.. (2021). *Akkermansia muciniphila* secretes a glucagon-like peptide-1-inducing protein that improves glucose homeostasis and ameliorates metabolic disease in mice. Nat. Microbiol. 6, 563–573. doi: 10.1038/s41564-021-00880-5, PMID: 33820962

[ref89] Yu J. S. Youn G. S. Choi J. Kim C. H. Kim B. Y. Yang S. J.. (2021). *Lactobacillus lactis* and *Pediococcus pentosaceus*-driven reprogramming of gut microbiome and metabolome ameliorates the progression of non-alcoholic fatty liver disease. Clin. Transl. Med. 11:e634. doi: 10.1002/ctm2.634 34965016 PMC8715831

[ref90] Yuille S. Reichardt N. Panda S. Dunbar H. Mulder I. E. (2018). Human gut bacteria as potent class I histone deacetylase inhibitors *in vitro* through production of butyric acid and valeric acid. PLoS One 13:e0201073. doi: 10.1371/journal.pone.0201073, PMID: 30052654 PMC6063406

[ref91] Zelante T. Iannitti R. G. Cunha C. De Luca A. Giovannini G. Pieraccini G.. (2013). Tryptophan catabolites from microbiota engage aryl hydrocarbon receptor and balance mucosal reactivity via interleukin-22. Immunity 39, 372–385. doi: 10.1016/j.immuni.2013.08.003, PMID: 23973224

[ref92] Zhai R. Xue X. Zhang L. Yang X. Zhao L. Zhang C. (2019). Strain-specific anti-inflammatory properties of two *Akkermansia muciniphila* strains on chronic colitis in mice. Front. Cell. Infect. Microbiol. 9:239. doi: 10.3389/fcimb.2019.00239, PMID: 31334133 PMC6624636

[ref93] Zhang X. Chen S. Zhang M. Ren F. Ren Y. Li Y.. (2021). Effects of fermented milk containing *Lacticaseibacillus paracasei* strain shirota on constipation in patients with depression: a randomized, double-blind, placebo-controlled trial. Nutrients 13:2238. doi: 10.3390/nu13072238, PMID: 34209804 PMC8308326

[ref94] Zhao Y. Liu Q. Hou Y. Zhao Y. (2022). Alleviating effects of gut micro-ecologically regulatory treatments on mice with constipation. Front. Microbiol. 13:956438. doi: 10.3389/fmicb.2022.956438 36016793 PMC9396131

[ref95] Zhou Q. Costinean S. Croce C. M. Brasier A. R. Merwat S. Larson S. A.. (2015). MicroRNA 29 targets nuclear factor-κB–repressing factor and Claudin 1 to increase intestinal permeability. Gastroenterology 148:e158, 158–169.e8. doi: 10.1053/j.gastro.2014.09.037 PMC4303568 25277410

[ref96] Zhou Q. Pang G. Zhang Z. Yuan H. Chen C. Zhang N.. (2021). Association between gut Akkermansia and metabolic syndrome is dose-dependent and affected by microbial interactions: a cross-sectional study. Diabetes Metab. Syndr. Obes. 14, 2177–2188. doi: 10.2147/DMSO.S311388, PMID: 34040404 PMC8139944

[ref97] Zhuang M. Shang W. Ma Q. Strappe P. Zhou Z. (2019). Abundance of probiotics and butyrate-production microbiome manages constipation via short-chain fatty acids production and hormones secretion. Mol. Nutr. Food Res. 63:1801187. doi: 10.1002/mnfr.201801187, PMID: 31556210

