# Supplementary material for: Multi-strain probiotics alleviate loperamide-induced constipation by adjusting the microbiome, serotonin, and short-chain fatty acids in rats
Source: Front Microbiol. 2023 Jun 2;14:1174968. doi: 10.3389/fmicb.2023.1174968 (PMC10272585; doi:10.3389/fmicb.2023.1174968)
Supplement: Supplementary file 1 [file Data_Sheet_1.pdf]

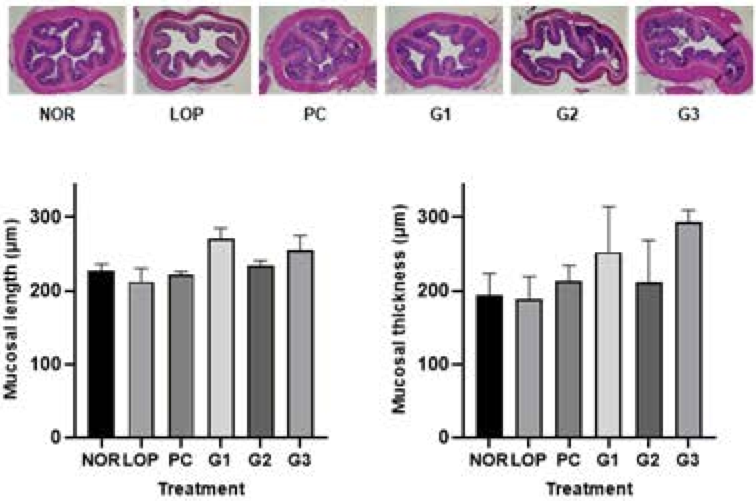

Supplementary Figure S1. Intestinal mucosal length and thickness after multi-strain probiotics Consti-Biome administration. The upper photograph shows the stained intestinal mucosa. The left graph shows the length of the mucosa and the right shows the thickness of the mucosa.

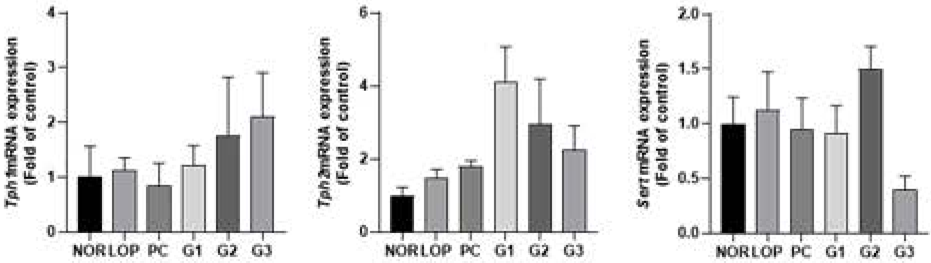

Supplementary Figure S2. mRNA expression related to serotonin according to constipation induction and multi-strain probiotics Consti-Biome treatment. mRNA expression level in the colon of normal control (NOR), loperamide administration (LOP), positive control (PC), low concentration multi-strain probiotics administration (G1, 2×10^8 cfu/ml), medium concentration multi-strain probiotics administration (G2, 2×10^9 cfu/ml), and high concentration probiotic administration (G3, 2×10^{10} cfu/ml) groups were measured 2 weeks after the multi-strain probiotics' treatment. Data are mean ± SEM of 7 animals in *Tph1*, *Tph2* and *Sert*.

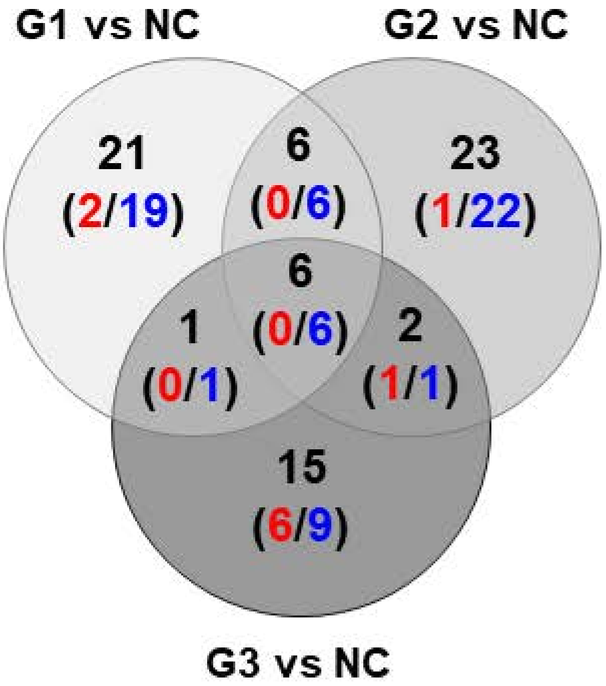

Supplementary Figure S3. The metabolites that show specific or common abundance pattern in multi-strain probiotics treatment groups compared to loperamide administration (LOP) group. The significantly difference of comparing from the LOP as per Mann-whitney U test. Red color means up-regulation at treatment group and Blue color means down-regulation at treatment group compared to LOP group, respectly.

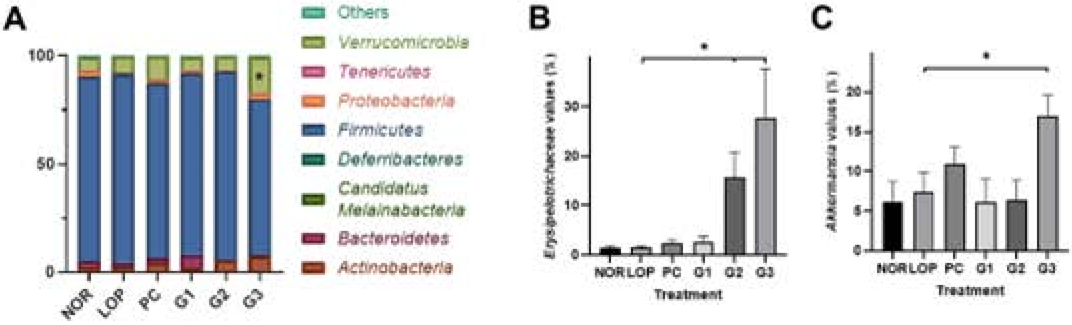

Supplementary Figure S4. Microbiome changes by induction of constipation and intake of multi-strain probiotics Consti-Biome. A is the representative phylum increase and decrease. B and C are variations of the family *Erysipelotrichaceae* and the genus *Akkermansia*, respectively. Microbiome of normal control (NOR), loperamide administration (LOP), positive control (PC), low concentration multi-strain probiotics administration (G1, 2×10^8 cfu/ml), medium concentration multi-strain probiotics administration (G2, 2×10^9 cfu/ml), and high concentration probiotic administration (G3, 2×10^{10} cfu/ml) groups were analyzed with fecal samples 2 weeks after treatment with multi-strain probiotics. Data are mean ± SEM of 5 animals in B and C. $^*p < 0.05$, significantly different from the LOP as per unpaired t test.

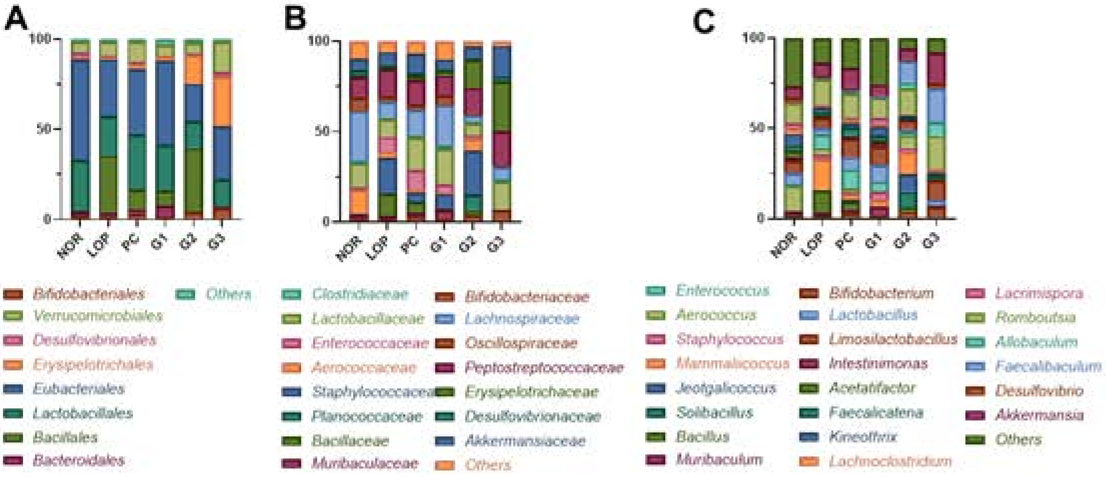

Supplementary Figure S5. Microbiome changes by induction of constipation and intake of multi-strain probiotics Consti-Biome. A, B, and C represent the order, the family, and the genus, respectively. Microbiome of normal control (NOR), loperamide administration (LOP), positive control (PC), low concentration multi-strain probiotics administration (G1, 2×10^8 cfu/ml), medium concentration multi-strain probiotics administration (G2, 2×10^9 cfu/ml), and high concentration probiotic administration (G3, 2×10^{10} cfu/ml) groups were analyzed with fecal samples 2 weeks after treatment with multi-strain probiotics.

Supplementary Table 1. Summary of univariate statistics on G1 vs LOP

Mouse cecal metabolite (significantly different in G1)	Fold change
2'-Deoxyuridine	5.1744
L-Aspartic acid	1.8973
3-Hydroxyanthranilic acid	0.5313
Choline	0.5242
Gluconic acid	0.5044
D-(-)-Ribose	0.4811
3-Methylglutaric acid	0.4748
N3,N4-Dimethyl-L-arginine	0.4624
Skatole	0.4600
L-Serine	0.4518
DL-Tryptophan	0.4465
Creatine	0.4388
Cholecalciferol	0.4383
(2R)-2,3-Dihydroxypropanoic acid	0.4316
6-Methylquinoline	0.4268
L-Phenylalanine	0.4167
2'-O-Methylguanosine	0.3883
Oleoyl ethanolamide	0.3807
4'-Methoxyacetophenone	0.3787
Cytidine	0.3771
Uracil	0.3768
D-Sphingosine	0.3482
Xanthurenic acid	0.3475
N,N-Dimethylsphingosine	0.2759
Methenolone	0.2738
DL-Malic acid	0.2158
Salicylic acid	0.2102
Fumaric acid	0.2043
Palmitoylcarnitine	0.1754
N,N-Diethylethanolamine	0.1524
Citric acid	0.1391
Creatinine	0.0580
Leucine	0.0271
L-Valine	0.0038

P-value were calculated based on Mann-whitney u-test

Supplementary Table 2. Summary of univariate statistics on G2 vs LOP

Mouse cecal metabolite (significantly different in G2)	Fold change
3-indole butyric acid	16.9055
Isoferulic acid	2.6747
DL-Lysine	0.7242
N-Acetylneuraminic acid	0.6880
Pipecolic acid	0.6634
β-Alanine	0.6116
Trigonelline	0.6039
β-D-Glucopyranuronic acid	0.5954
Choline	0.5269
L-(+)-Citrulline	0.5172
5-Aminovaleric acid	0.5166
Desoxycortone	0.5111
L-(+)-Lactic acid	0.4969
DL-Carnitine	0.4957
Glycerophospho-N-palmitoyl ethanolamine	0.4422
6-Hydroxycaproic acid	0.4351
Pantothenic acid	0.4300
(2R)-2,3-Dihydroxypropanoic acid	0.4198
L-(-)-Methionine	0.4178
N-Acetyl-D-alloisoleucine	0.4172
N3,N4-Dimethyl-L-arginine	0.4166
3-Methylglutaric acid	0.4137
L-Cysteinesulfinic acid	0.4036
Uracil	0.3950
5α-Pregnan-3,20-dione	0.3923
Nicotinic acid	0.3862
Gluconic acid	0.3651
2'-O-Methylguanosine	0.3492
δ-Gluconic acid δ-lactone	0.3388
Xanthine	0.3333
Spermine	0.3173
Oleoyl ethanolamide	0.2555
Fumaric acid	0.2170
DL-Malic acid	0.2165
Xanthurenic acid	0.1829
(+/-)11-HETE	0.1541
Citric acid	0.1091

P-value were calculated based on Mann-whitney u-test

Supplementary Table 3. Summary of univariate statistics on G3 vs LOP

Mouse cecal metabolite (significantly different in G3)	Fold change
Isoferulic acid	3.9411
L-Histidine	3.8110
D-(-)-Glutamine	2.7685
Butyrate	1.9731
Valerate	1.7327
Propionate	1.7179
Acetate	1.4719
3-Hydroxyanthranilic acid	0.5003
Methylimidazoleacetic acid	0.4794
Suberic acid	0.4259
Gluconic acid	0.4181
3-Methylglutaric acid	0.3871
Fumaric acid	0.3302
16-Hydroxyhexadecanoic acid	0.3155
Spermine	0.3084
Uracil	0.3062
Quinolinic acid	0.2862
Xanthurenic acid	0.2502
Cytosine	0.2412
Phenylpyruvic acid	0.2179
Kynurenic acid	0.2159
Xanthosine	0.1705
2'-O-Methylguanosine	0.1522
2-Oxoglutaric acid	0.1000

P-value were calculated based on Mann-whitney u-test

Supplementary Table 4. Summary of statistics of short chain fatty acids

**Mouse cecal metabolite**	
**Short chain fatty acids (SCFAs)**	**p-value**
Acetic acid	0.097
Propionic acid	0.091
Butyric acid	0.142
Iso-butyric acid	0.279
Valeric acid	0.254
Iso-valeric acid	0.338

P-value were calculated based on Kruskal-Wallis test.
